# Artificial intelligence-based expert trajectory guidance in an *ex vivo* robot-assisted renal wound suturing training model

**DOI:** 10.3389/fsurg.2026.1874149

**Published:** 2026-07-02

**Authors:** Tailai Zhou, Tongyu Jia, Shangwei Li, Jiachen Zheng, Haotian Hou, Houming Zhao, Jichen Wang, Ji Feng, Xin Ma

**Affiliations:** 1 Senior Department of Urology, Chinese PLA General Hospital, Beijing, China; 2 Department of Urology, The Third Medical Center of Chinese PLA General Hospital, Beijing, China

**Keywords:** artificial intelligence, renal wound suturing, robot-assisted partial nephrectomy, surgical skill acquisition, surgical training, surgical trajectory guidance

## Abstract

**Introduction:**

Renorrhaphy is one of the most technically demanding steps in robot-assisted partial nephrectomy, requiring expert-level suturing to ensure adequate hemostasis and long-term renal function preservation, yet acquiring such proficiency requires extensive practice under expert supervision. Although artificial intelligence has been increasingly applied to perioperative surgical care, its potential to learn expert operative patterns from standard surgical video and translate them into real-time visual guidance for surgical training remains largely unexplored. Here we developed and evaluated an artificial intelligence framework that learns expert suturing trajectories from standard endoscopic video and provides intraoperative visual guidance for renal wound suturing training.

**Methods:**

A multicenter expert trajectory dataset was constructed from robot-assisted partial nephrectomy procedures performed at two medical centers, supported by a standardized data pipeline comprising 28-class scene annotation, temporal phase labeling of complete suturing actions, and sliding-window trajectory sampling. Building on these data, we developed a Scene-Aware Transformer that integrates instrument motion with surgical scene context to predict future trajectories, and prospectively evaluated the resulting guidance system in a pilot *ex vivo* porcine kidney feasibility training study involving 24 novice trainees.

**Results:**

The dataset comprised 18,515 annotated frames, 806 complete suturing actions, and 24,897 valid trajectory samples. On the independent held-out institutional test set, the model achieved an average displacement error of 34.25 pixels and a final displacement error of 52.54 pixels, with an end-to-end inference latency of 32.7 ms under laboratory computational conditions. In the prospective training study, novice trainees who received expert trajectory guidance significantly outperformed the unguided control group across six of eight performance measures by the final assessment, and maintained their advantage during unguided evaluation sessions.

**Discussion:**

These preliminary findings suggest that artificial intelligence-derived expert trajectory guidance may support short-term skill acquisition for renal wound suturing. As the prospective training component was a single-institution feasibility study without long-term retention or clinical transfer assessment, larger multicenter randomized trials are warranted before broader integration into surgical training curricula.

## 1 Introduction

Robotic minimally invasive surgery continues to expand across clinical domains, with emerging robotic platforms increasingly emphasizing specialized, flexible, and image-guided applications (1, 2). In parallel, artificial intelligence (AI) has shown substantial promise across the surgical workflow in recent years, including preoperative three-dimensional reconstruction (3), critical view of safety assessment, automated skill evaluation (4) and AI-based surgical coaching (5). However, most of these advances have been largely restricted to automated recognition and retrospective assessment of surgical behavior. The application of deep learning to learn expert motion patterns and provide real-time operative guidance remains largely unexplored. This gap is particularly relevant in surgical training, where novice surgeons require intuitive, spatial references to facilitate the acquisition of complex procedural skills (6).

The use of expert trajectories as the reference standard is supported by previous work. Multiple studies have demonstrated that expert surgeons exhibit significantly shorter path lengths, smoother instrument movements, and greater economy of motion compared with novices (7). However, kinematics-based methods depend on proprietary robotic data streams, limiting generalizability beyond specific platforms (8). Recent work on hybrid optical-vision tracking in laparoscopy further illustrates the relevance of video-based tracking for surgical navigation and reconstruction, supporting the broader technical rationale for deriving instrument coordinates from standard surgical video (9). Likewise, conventional trajectory prediction models such as Social LSTM (10), Social GAN (11), and Trajectron++ (12) were designed for pedestrian forecasting under multi-agent social interaction assumptions and are not well suited to the constrained instrument-tissue interaction in surgery. More recently, Dou et al. proposed the expert trajectory prediction framework for endoscopic submucosal dissection surgery using equivariant diffusion, but their study remained limited to algorithmic validation without downstream applications (13). Collectively, these studies highlight the potential of data-driven surgical motion modeling, but translation into procedure-specific visual guidance for complex reconstructive tasks remains limited. To our knowledge, video-derived expert trajectory guidance has not yet been specifically evaluated for robotic renorrhaphy training.

This unmet need is particularly relevant in renorrhaphy during robot-assisted partial nephrectomy (RAPN). Renorrhaphy requires precise control of needle angle, bite depth, tissue approximation, and suture tightness under warm ischemia constraints (14, 15), and the quality of wound closure directly influences intraoperative blood loss, postoperative complications, and long-term renal functional preservation (16, 17). In this setting, expert trajectory guidance may provide novices with an intuitive spatial reference for instrument direction, needle entry and exit path, and motion economy, thereby potentially improving not only task efficiency but also tissue handling, depth control, and final closure quality. Previous studies have reported that the robot-assisted partial nephrectomy (RAPN) learning curve generally requires at least 50 cases, with wound reconstruction consistently recognized as the principal technical bottleneck (18).

To address these challenges, we developed a deep learning framework for expert-guided surgical trajectory prediction and visual guidance in renorrhaphy. First, we constructed a multicenter expert trajectory dataset with a systematic data generation pipeline for renal wound suturing, which includes surgical scene annotation, suturing action labeling, and sliding-window trajectory sampling, providing a reusable data foundation and methodological reference for future research. Second, we developed a video-based prediction model integrating instrument motion and visual scene context to generate future trajectories for surgical visual guidance. Third, we evaluated this approach in a prospective pilot training study to explore its potential educational value in surgical skills training.

## 2 Materials and methods

### 2.1 Data collection

**Figure 1:**
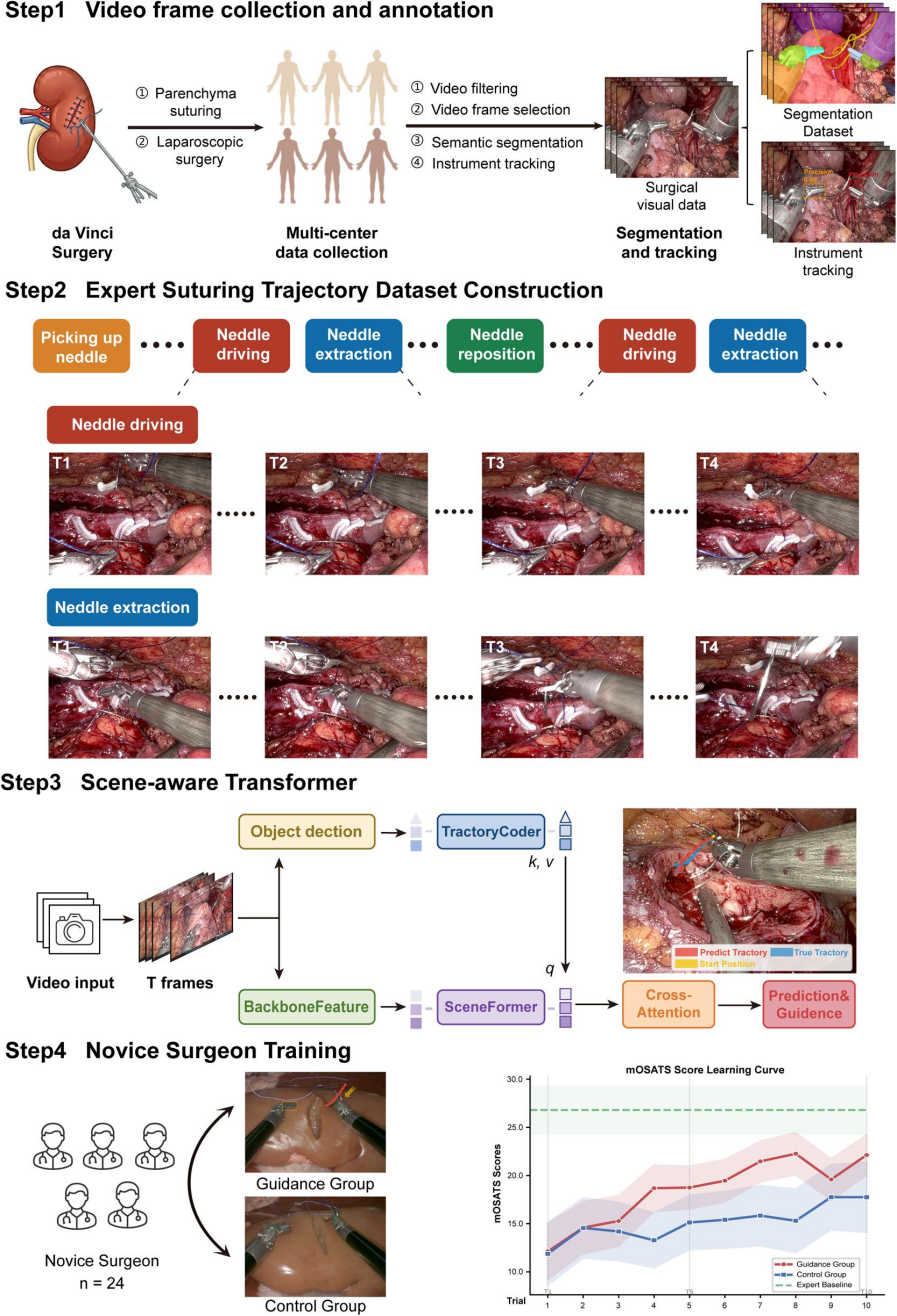
Overall study workflow. Step 1, surgical videos from RAPN were collected from multiple centers to establish the visual scene dataset. Step 2, continuous suturing sequences were decomposed into key suturing actions and were further sampled into trajectory segments to construct the expert suturing trajectory dataset. Step 3, a scene-aware Transformer framework was developed for future trajectory prediction and suturing visual guidance. Step 4, left figure, the proposed visual guidance was evaluated in a prospective novice surgeon training. In the surgical field view, red line denotes the predicted trajectories, and yellow arrows indicate the directional guidance generated from the start and end points of the predicted trajectories. Right figure, comparison between guided and control groups to assess learning-curve improvement.

The overall study workflow is shown in Figure 1. Surgical video data from RAPN were collected between May and December 2024 at The Third Medical Center and First Medical Center of the Chinese PLA General Hospital. Inclusion criteria were age 18 years or older, an imaging-confirmed renal mass with an indication for partial nephrectomy, and availability of a complete intraoperative video recording. Exclusion criteria included intraoperative conversion to radical nephrectomy, interrupted recording, and poor surgical field visibility. The final dataset comprised 50 RAPN procedures (Supplementary Table S1). A total of 45 patients from the Third Medical Center were used for model development (40 training, 5 validation), and 5 patients from the First Medical Center served as an independent, held-out institutional test set. All dataset splitting was performed at the patient/procedure level, such that all frames, sub-segments, and trajectory samples derived from the same procedure were assigned exclusively to one split to prevent overlap of samples across the training, validation, and test sets. All procedures were performed by 5 senior urologists, each with more than 15 years of surgical experience and more than 200 RAPN cases, using the da Vinci Xi Surgical System (Intuitive Surgical Inc). Intraoperative videos were recorded at 1080i and 60 frames per second.

### 2.2 Scene annotation and trajectory dataset construction

A systematic data pipeline was established for model development. To begin, a 28-class taxonomy was defined for surgical instruments and anatomical structures within the suturing scene, including the clasper, wrist, and shaft of the left and right robotic needle drivers; the suturing needle and thread; and relevant anatomical structures such as the renal wound, renal parenchyma, and resected tumor specimen (Supplementary Figure S1A). Nine trained annotators labeled all visible instruments, surgical accessories, and anatomical structures using the Medical Imaging Interaction Toolkit (MITK, version 2024.12). Based on this taxonomy, pixel-level semantic segmentation was performed on a representative subset of video frames sampled from suturing segments. These annotations were used to fine-tune a YOLOv13 (19) object detection model for automated instrument localization and visual scene feature extraction. For trajectory construction, the primary coordinate input was derived from the detected robotic instrument clasper, whereas the broader 28-class scene taxonomy was used to provide contextual visual features for the Scene-Aware branch.

Subsequently, two experienced urologists independently reviewed all suturing segments and decomposed complete suturing actions into alternating needle-driving and needle-extraction phases. Each action was further subdivided into sub-segments to account for interruptions caused by needle re-grasping or changes in field of view. To increase the sample size, a sliding-window sampling strategy was applied within each annotated sub-segment. Each window contained 20 key frames sampled at an interval of 5 frames and advanced with a stride of 1 frame (i.e., one key-frame extracted every 5 frames), yielding an 8-frame observation horizon and a 12-frame prediction horizon. The fine-tuned YOLOv13x model was then used to obtain the 2-dimensional coordinates of the operating instrument clasper.

### 2.3 Validation of YOLO-based trajectory annotation against manual annotation

To assess the reliability of YOLO-based automatic trajectory annotation, an independent manual annotation was performed on the same set of surgical videos used in this study. Two trained annotators independently labeled the needle-grasping point at the clasper as the anatomical reference because it most directly represents the operative landmark a surgeon visually tracks and provides a single, unambiguous, and reproducible visual target on each clasper.

For each trajectory, 9 evenly spaced keyframes were sampled from the start to the end of the suturing sub-segment to cover the key phases of each suturing action while keeping the manual labeling cost feasible (13). YOLO-derived bounding-box centers on the same 9 keyframes were then compared with the corresponding manual annotations. Five complementary metrics were used to quantify agreement between YOLO-derived and manually annotated trajectories: raw localization error (Raw LE), defined as the frame-level Euclidean distance between YOLO bounding-box centers and manual annotations; residual localization error (Residual LE), defined as the same distance after removing the per-trajectory systematic offset between the bounding-box center and the needle-grasping point, which isolates random YOLO detection noise from the constant geometric difference between the two landmarks; discrete Fréchet distance; Hausdorff distance; and trajectory-length error. Full metric definitions are provided in Supplementary Methods S1.

### 2.4 Annotation quality control

Before formal annotation, all annotators received standardized training. Annotation qualification was assessed using pre-labeled samples derived from real surgical videos, and participation in formal annotation required pairwise inter-annotator agreement greater than 0.80 across all annotator pairs (20, 21). For semantic segmentation, inter-annotator agreement was quantified using the mean intersection over union (mIoU) across all 28 categories. For phase annotation, inter-annotator agreement was quantified using temporal intersection over union (tIoU) between annotators on the same pre-labeled segments.

During formal annotation, inter-annotator agreement was evaluated in a randomly sampled 10% subset of the annotated dataset. Inter-annotator mIoU for semantic segmentation was 0.89 ± 0.06, and inter-annotator tIoU for phase annotation was 0.93 ± 0.04, indicating high consistency across both tasks.

### 2.5 Scene-aware transformer (SAT) development

The proposed framework adopts a dual-branch encoder-decoder architecture to predict future instrument trajectories over subsequent key frames. The trajectory branch captures instrument motion dynamics by encoding the 2-dimensional coordinate differences between adjacent key frames as normalized velocity vectors, which are projected through a linear embedding layer, combined with positional encoding, and processed by a Transformer encoder to generate a trajectory memory. In parallel, the Scene-Aware branch extracts visual context from the surgical field. For each observed frame, visual feature vectors are obtained from the fine-tuned and frozen YOLOv13 backbone and processed by a scene encoder to model temporal scene evolution within the observation window.

The outputs of the trajectory and scene encoders are integrated through a cross-attention module, enabling each time step in the trajectory representation to attend selectively to the most relevant visual context. The Transformer decoder then autoregressively generates a future velocity sequence, which is cumulatively summed to recover predicted position coordinates. Detailed architectural specifications, mathematical formulation, and hyperparameter settings are provided in the Supplementary Methods S2.

### 2.6 Training study design and participants

A prospective pilot training study was conducted to evaluate the utility of AI-based visual trajectory guidance for novice surgeons acquiring renal wound suturing skills. Before the training study, the Scene-Aware Transformer model was transfer fine-tuned using 20 previously collected *ex vivo* porcine kidney suturing videos to adapt to the visual and motion characteristics of the training environment (Supplementary Methods S3). Because no prior effect size estimates were available for this novel guidance approach, the study was designed as a feasibility investigation to generate efficacy data for future adequately powered trials. As a simulation-based educational study conducted exclusively on *ex vivo* animal tissue without patient involvement, it was not considered a clinical trial under ICMJE criteria.

Twenty-four postgraduate surgical trainees with fewer than 10 cumulative hours of laparoscopic experience were recruited from the Chinese PLA General Hospital. Before formal training, all participants completed a standardized preparatory curriculum consisting of didactic instruction on robotic laparoscopic techniques and hands-on robotic instrument handling, followed by competency assessment on silicone practice modules. Participants were then allocated by computer-generated numbers to a guidance group (*n* = 12), which received visual overlays of expert trajectories during practice, or a control group (*n* = 12), which underwent equivalent training without visual guidance. Baseline characteristics, including sex, age, postgraduate year, and prior laparoscopic experience, did not differ between groups (Supplementary Table S2). Group allocation was generated by computer-generated random numbers and concealed from outcome assessors. Six experienced urologists were invited to perform the same task to establish an expert baseline and were independent from the trajectories used for model training.

### 2.7 Training procedure

Fresh porcine kidneys obtained from a local abattoir were used as the *ex vivo* training model. Each kidney was mounted on a fresh porcine rib to simulate the RAPN surgical field setting. Before each trial, a standardized renal wound model was created measuring 35 mm in length, 10 mm in width, and 5 mm in depth, with a maximum allowable deviation of 1 mm in depth and width, 2 mm in length.

Training was conducted over 7 days, with study sessions on days 1, 3, 5, and 7. Each participant completed 10 independent suturing trials: 3 trials on days 1, 3, and 5, and 1 trial on day 7, with a 30-minute rest interval between consecutive trials. Trials 1, 5, and 10 were designated as baseline, mid-training, and post-training assessment sessions, respectively. In these assessment sessions, both groups performed the task independently without visual guidance to ensure that outcomes reflected true technical performance. The remaining 7 trials (trials 2–4 and 6–9) served as practice sessions. During these practice sessions, the guidance group received overlaid expert trajectory prediction lines within the surgical field, whereas the control group underwent otherwise identical training without visual overlays. The visual guidance was rendered as a continuously updated overlay on the endoscopic console view. At each prediction step, the predicted expert trajectory was displayed as a red line connecting the 12 predicted future clasper positions, with a yellow directional arrow indicating the overall movement direction derived from the start and end points of the predicted path. The overlay was refreshed as new instrument coordinates and visual features became available. To minimize visual clutter and avoid misleading cues, only the single most recent predicted trajectory was shown at any time. Prediction uncertainty was not displayed in the current feasibility system, and the overlay was rendered as a semi-transparent thin line so as not to obscure the underlying tissue and needle.

### 2.8 Outcome measures

Surgical performance was assessed using 8 measures: suturing time, defined as elapsed time from initiation to completion of wound closure; time per stitch; instrument-needle interaction count; maximum gap length; total gap length; wound tear count; error frequency (defined as the total number of failed suturing attempts requiring repetition because of an incorrect needle angle, movement direction, or instrument trajectory); and modified Objective Structured Assessment of Technical Skills (mOSATS) score (22), a composite measure of surgical technical skill adapted from the original OSATS framework for the present task (23), comprising 6 domains each rated on a 1 to 5 Likert scale (Supplementary Table S3).

Maximum and total gap length were measured immediately after each trial using a digital caliper. The mOSATS rubric was adapted for the present robotic renal wound suturing task to capture procedure-specific domains such as needle handling, tissue approximation, suture tension, instrument coordination, and overall flow. Although the mOSATS framework has been used in prior surgical skills research (22), the task-specific adaptation used in this study has not been independently validated for robotic renal wound suturing and was therefore interpreted as an exploratory educational outcome. All remaining measures were jointly assessed by 2 senior urologists through blinded video review with disagreements resolved by consensus. The 2 outcome assessors were not involved in the training intervention, were independent of the expert baseline surgeons, and were blinded to group allocation and trial order during video review. The intraclass correlation coefficient for the mOSATS total score was 0.87 (95% CI, 0.82-0.93).

### 2.9 Statistical analysis

Trajectory prediction performance was assessed using three complementary metrics: Average Displacement Error (ADE), defined as the mean Euclidean distance between the predicted and ground-truth positions across all prediction time steps; Final Displacement Error (FDE), defined as the Euclidean distance between the predicted and ground-truth positions at the final prediction time step; and Discrete Fréchet Distance (FD), which quantifies the overall shape similarity between the predicted and ground-truth trajectories (metric definitions in Supplementary Methods S2). All metrics were calculated directly in pixel space on the 1,920 × 1,080 image plane and model comparisons were conducted on the independent held-out test set.

For the training study, between-group comparisons at the prespecified unguided assessment sessions (Trials 1, 5, and 10) were performed using the two-sided Mann–Whitney *U*-test, and within-group longitudinal changes between assessment sessions were evaluated using the Wilcoxon signed-rank test. Because this was a pilot feasibility study with multiple exploratory outcomes and a limited sample size, *P* values were interpreted as exploratory rather than confirmatory. Benjamini-Hochberg false-discovery-rate correction was applied across the eight outcomes within each assessment time point as a sensitivity analysis. A *post hoc* sensitivity analysis indicated that, with 12 participants per group, a two-sided alpha level of 0.05, and 80% power. Effect sizes were quantified using Cohen's d and rank-biserial correlation. Continuous variables are presented as mean ± standard deviation (SD), categorical variables as frequencies, and trial-by-trial learning curves across the 10 training trials were summarized descriptively using group-level means and SDs.

## 3 Results

### 3.1 Dataset characteristics and detection model

The dataset comprised 50 RAPN procedures from two medical centers totaling approximately 9.7 h of suturing video (mean duration, 13.7 ± 3.4 min per patient). A representative subset of 18,515 frames sampled from suturing segments in the training set underwent pixel-level semantic segmentation annotation and was used to support automated instrument localization and scene feature extraction by the front-end YOLOv13 model. The fine-tuned YOLOv13x detection model achieved high accuracy for key surgical instruments: mAP50 exceeded 0.96 for both left and right robotic needle driver claspers and was 0.920 for the suturing needle on the independent test set (Supplementary Table S4). Detection performance varied across the 28 semantic categories, with lower accuracy observed for rare or visually heterogeneous classes such as accessory laparoscopic instruments, bulldog appliers, or resected tumor specimens. However, these classes were not used as direct trajectory-coordinate targets. The trajectory prediction task primarily depended on reliable localization of the robotic needle-driver clasper, for which detection performance was high, while the remaining classes contributed auxiliary scene-context information. Heterogeneity in scene-category detection may still affect the quality of contextual visual features and is therefore considered a potential source of residual model uncertainty.

### 3.2 Reliability of YOLO-based trajectory annotation

Of the 1,000 manually annotated trajectories, visual inspection of representative trajectories spanning both high and low localization errors confirmed that YOLO-derived and manually annotated trajectories tracked the same motion pattern, differing primarily by a constant geometric offset between the bounding-box center and the needle-grasping point (Supplementary Figures S2A,B). After removing this per-trajectory systematic offset, the residual localization error was 12.36 ± 9.37 pixels (median, 10.22; Supplementary Figures S2C,D). Three additional shape-similarity metrics further supported agreement between the two annotation sources: Fréchet distance, 52.91 ± 21.82 pixels; Hausdorff distance, 52.37 ± 21.66 pixels; and trajectory-length error, 6.15% (Supplementary Figure S2E; Supplementary Table S5).

It should be noted that the Fréchet and Hausdorff distances were computed on raw trajectories without removing the systematic offset, and therefore reflect the constant geometric translation between the bounding-box center and the needle-grasping point (mean bias magnitude, 36.86 pixels). In contrast, the residual localization error (∼12 pixels) and the translation-invariant trajectory-length error (6.15%) directly demonstrate that YOLO-derived and manually annotated trajectories share nearly identical motion shapes, differing only by a fixed geometric offset. Together, these findings support the reliability of YOLO-based trajectory annotation as a scalable substitute for manual annotation in this task (Supplementary Table S5).

### 3.3 Trajectory dataset features

**Figure 2:**
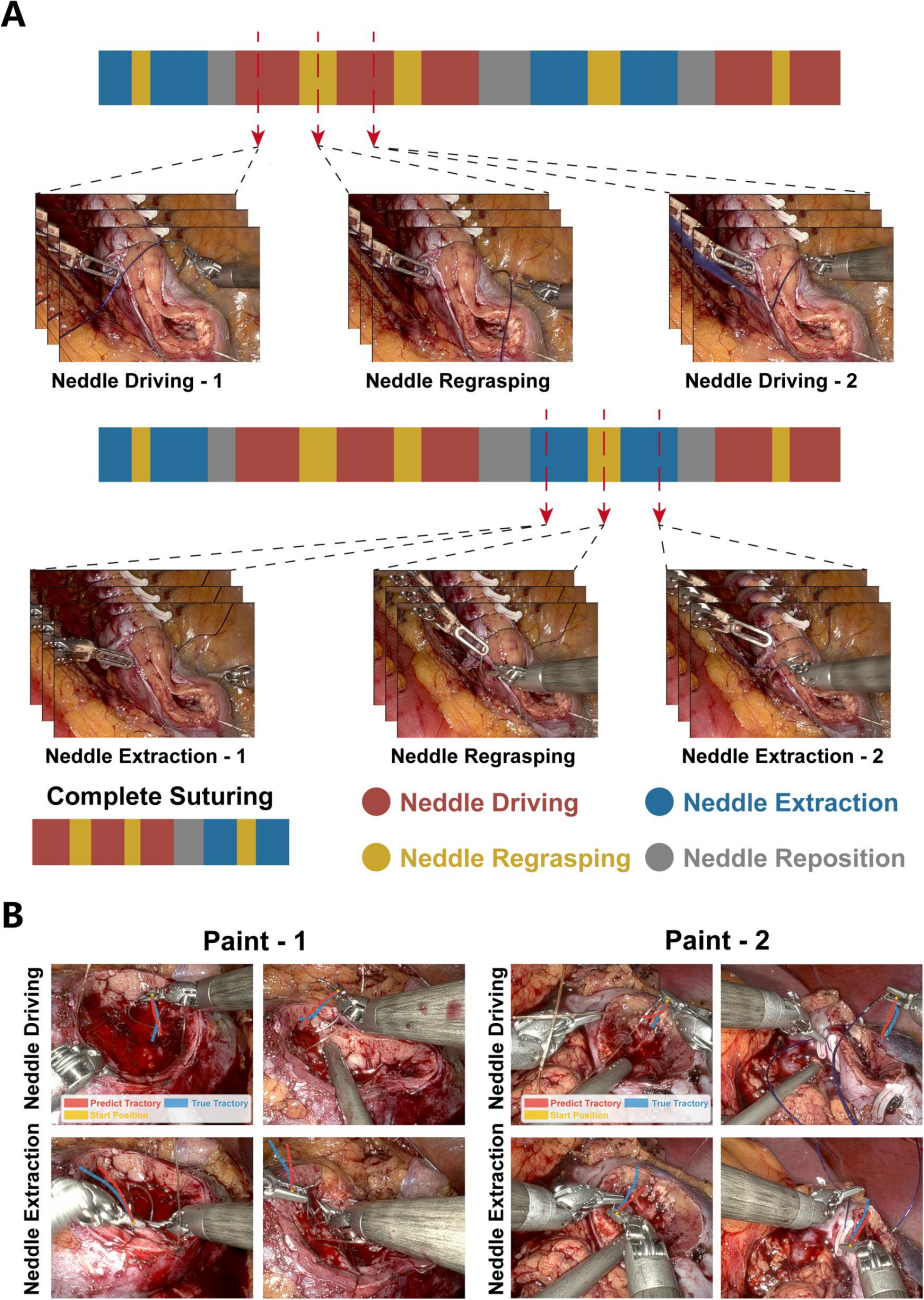
Expert suturing trajectory dataset construction and trajectory prediction visualizations. **(A)** Continuous suturing sequences were decomposed into alternating needle-driving and needle-extraction actions. Motion interruptions caused by needle regrasping or reposition were labeled separately, and continuous sub-segments were used for trajectory window sampling. **(B)** Representative examples of predicted and ground-truth trajectories.

For construction of the expert suturing trajectory dataset, each continuous suturing sequence was decomposed into complete suturing actions and further subdivided into needle-driving and needle-extraction sub-segments to account for interruptions caused by needle re-grasping or changes in field of view (Figure 2A).

Across 50 procedures, 806 complete suturing actions and 3,089 sub-segments were labeled. The training, validation, and test sets contained 646, 82, and 78 complete suturing actions (average 16.1 ± 4.2, 16.6 ± 2.8, and 15.6 ± 3.4 per patient). Needle-driving sub-segments were more frequent than needle-extraction sub-segments (40.2 ± 14.2 vs. 21.2 ± 6.0 per patient), reflecting the greater motion complexity and longer duration of the needle-driving action.

From action sub-segments, 24,897 valid trajectory samples were generated, including 70.8 ± 48.6 pixels in the observation segment and 130.6 ± 67.8 pixels in the prediction segment (Supplementary Figures S3A,B). The operating instrument clasper exhibited a spatially concentrated distribution within the surgical field, with a mean normalized coordinate of (0.557, 0.387) relative to the image dimensions, predominantly in the upper-middle region of the endoscopic field, consistent with the typical suturing workspace.

### 3.4 Efficiency of the scene-aware transformer

**Figure 3:**
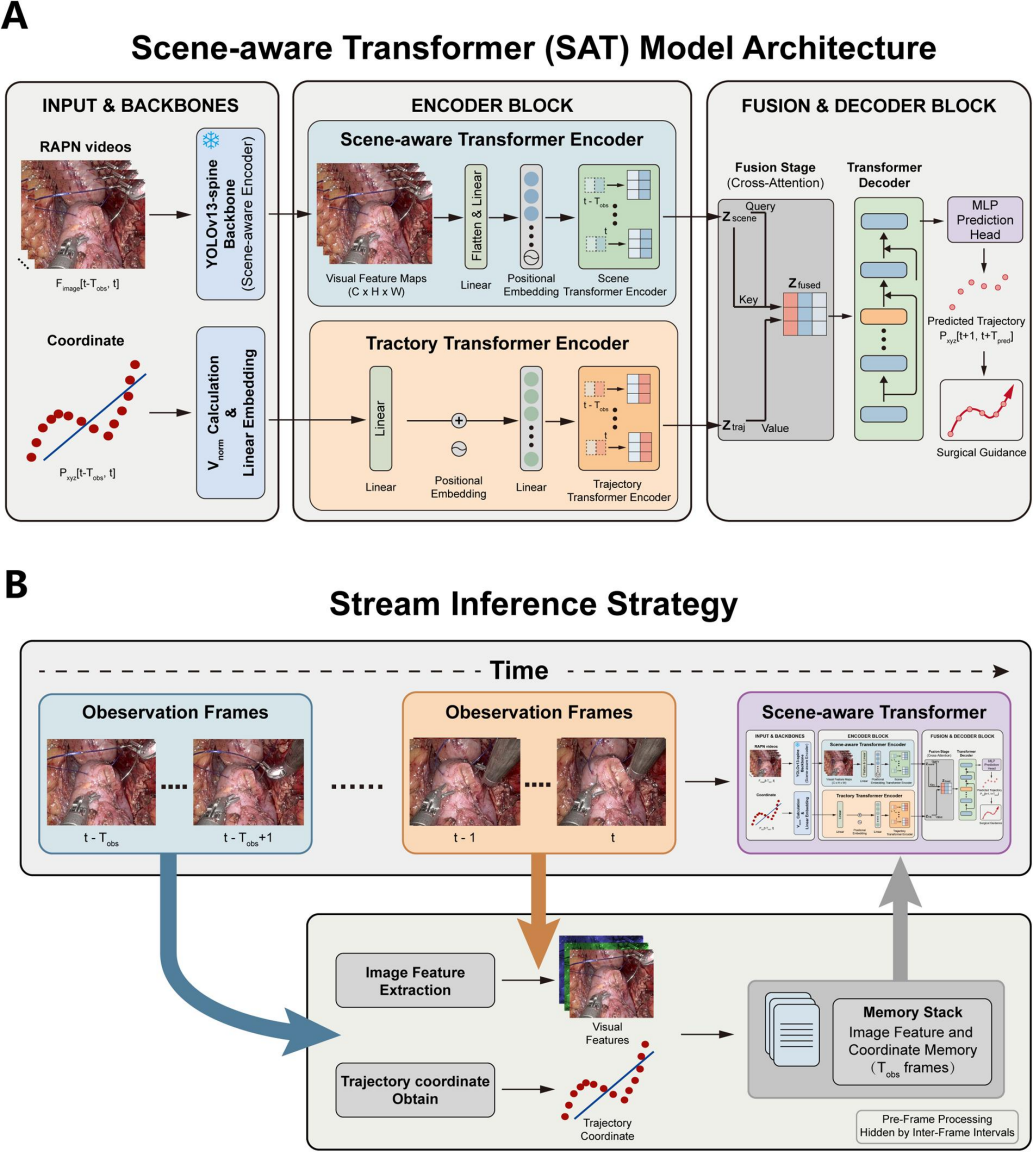
Scene-Aware transformer architecture and streaming inference strategy. **(A)** The model consisted of a scene-aware encoder for visual feature extraction, a trajectory encoder for historical motion modeling, cross-attention fusion, and a Transformer decoder for future trajectory prediction. **(B)** During streaming inference, visual features and trajectory coordinates were extracted online from incoming frames and cached in memory, allowing most preprocessing to be completed before the final observation frame.

The Scene-Aware Transformer adopts an encoder-decoder architecture with trajectory encoding branch and Scene-Aware branch to incorporate visual context into surgical instrument motion prediction (Figure 3A). The Scene-Aware Transformer trajectory prediction module contained 2.67 million trainable parameters, with only 0.35 million additional parameters in the Scene-Aware branch (13.1% of the prediction module). It should be noted that this parameter count refers specifically to the trajectory prediction module and does not include the YOLOv13 detector used for upstream instrument localization and visual feature extraction. Computational cost for the prediction module remained low, at 52.82 MFLOPs per forward pass, supporting efficient trajectory forecasting once instrument coordinates and visual features had been extracted. Additional ablation experiments comparing alternative fusion strategies showed that simple element-wise addition and concatenation improved performance over the trajectory-only baseline, while gated fusion provided further gains. Cross-attention achieved the best overall performance across ADE, FDE, and FD, reducing ADE, FDE, and FD by 14.31%, 16.15%, and 17.28%, respectively, compared with the trajectory-only baseline (Supplementary Table S6).

To satisfy the latency requirements for intraoperative assistance, we implemented a streaming inference strategy in which instrument detection and scene feature extraction were performed online as each video frame arrived and cached in memory (Figure 3B). On a platform equipped with an AMD Ryzen Threadripper 7960X CPU and an NVIDIA RTX 5,090 GPU, preprocessing required 15.3 ms per key frame and end-to-end latency for the final frame was 32.7 ms. It should be noted that these results demonstrate real-time feasibility under high-end laboratory computational conditions; however, performance on more typical operating-room hardware and integration into clinical video systems require further validation.

### 3.5 Prediction performance of the scene-aware transformer

On the independent test set, the Scene-Aware Transformer achieved an average displacement error (ADE) of 34.25 pixels and a final displacement error (FDE) of 52.54 pixels, with representative examples shown in Figure 2B. In ablation testing, addition of the Scene-Aware branch with cross-attention fusion improved ADE by 5.72 pixels (14.31%) and FDE by 10.41 pixels (16.53%) relative to the baseline model, supporting the contribution of visual scene context to trajectory prediction. Compared with conventional trajectory prediction approaches, the proposed model achieved competitive ADE and FDE performance with fewer parameters, highlighting its efficiency and predictive accuracy (12, 24–27) (Supplementary Table S6).

Prediction error increased with longer forecasting horizons, from a mean of 17.91 pixels at steps 1–4 to 48.56 pixels at steps 9–12, consistent with the expected error accumulation of autoregressive models. Interestingly, the Scene-Aware Transformer exhibited a slower error growth rate at longer prediction horizons than the baseline model, suggesting that scene information helped constrain prediction drift over time (Supplementary Figure S4A). Finally, the SAT model achieved favorable trajectory prediction performance across all five patients in the test set (Supplementary Figure S4B).

### 3.6 Training study outcomes

**Figure 4:**
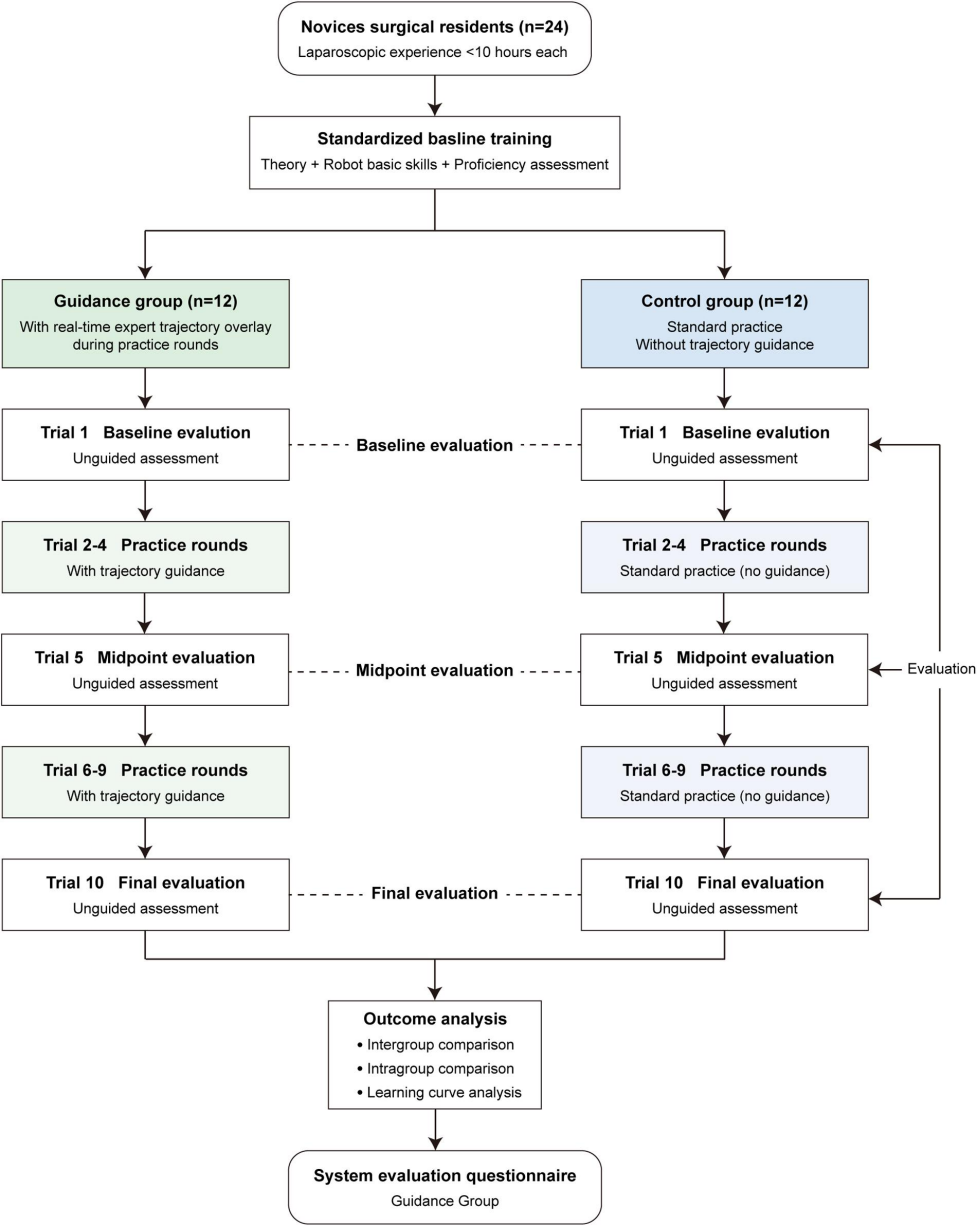
Prospective training study design. Schematic overview of the prospective training study comparing guided and unguided practice in novice surgeons.

**Figure 5:**
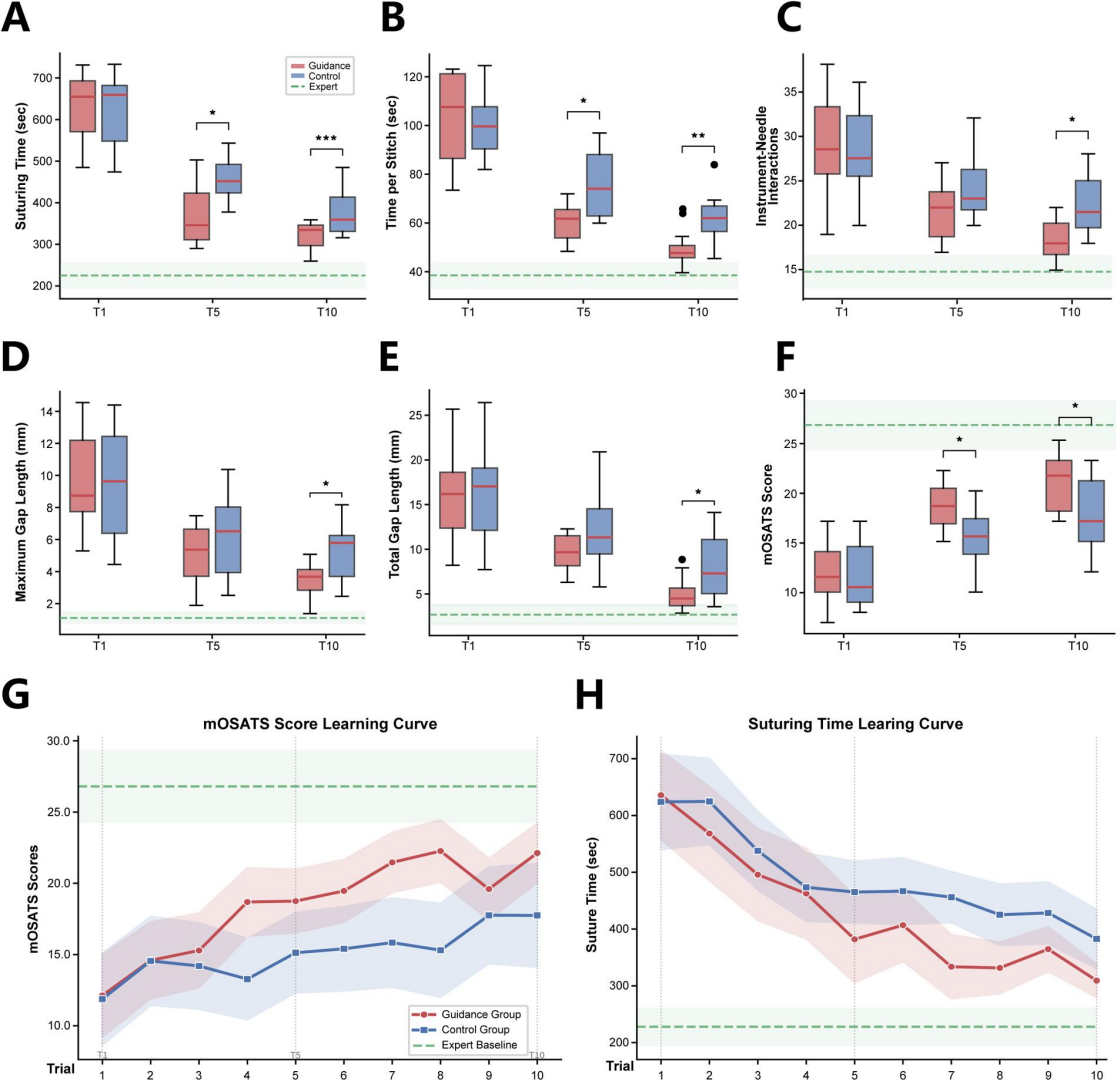
Comparative training outcomes and learning-curve analysis. **(A–F)** Surgical performance metrics at baseline, midpoint, and final assessment in the guidance and control groups, with expert baseline values shown for reference. **(G,H)** Learning curves for mOSATS score and suturing time across the 10 training trials. Guidance group, *n* = 12; control group, *n* = 12; expert baseline, *n* = 6. Expert baseline bands indicate mea*n* ± SD. *P < 0.05; ***P < 0.001.

The overall training design is shown in Figure 4. Among 24 trainees, no between-group differences were observed at baseline (Trial 1) for any outcome measure (all *P* > 0.05), and both groups performed consistently below the expert baseline, supporting good comparability after group allocation (Figures 5A–F; Supplementary Table S7).

By the mid-training assessment (Trial 5), the guidance group had already demonstrated superior performance in suturing time, time per stitch, and mOSATS score (*P* < 0.05), with favorable trends also observed for instrument-needle interaction count and post-closure wound gap measures. These findings suggest that the effect of visual trajectory guidance on operative efficiency and global technical performance emerged early during training.

At the post-training assessment (Trial 10), between-group differences were more pronounced. The guidance group outperformed the control group in 6 of 8 outcome measures: suturing time (mean, 310.4 vs. 382.5 s; Cohen d, 1.75), time per stitch (mean, 49.2 vs. 61.7 s per stitch; Cohen d, 1.42), instrument-needle interaction count (mean, 18.4 vs. 22.3; Cohen d, 1.39), maximum gap length (mean, 3.5 vs. 5.3 mm; Cohen d, 1.25), total gap length (mean, 5.1 vs. 8.3 mm; Cohen d, 1.05), and mOSATS score (mean, 20.9 vs. 17.7; Cohen d, 1.09) (all *P* < 0.05). Relative to the expert baseline, the guidance group reached 78.9% of the expert-level mOSATS score and required 1.38-fold the expert suturing time, whereas the corresponding values in the control group were 66.7% and 1.70-fold, respectively. Error frequency showed a nonsignificant favorable trend in the guidance group, whereas wound tear count reached zero in both groups at the final assessment, suggesting a potential floor effect. These findings indicate that visual trajectory guidance was primarily associated with improved operative efficiency and closure quality in this pilot study, while its effect on safety-related errors remains uncertain (Figures 5A–F; Supplementary Figures S4C,D; Supplementary Table S7).

### 3.7 Learning curve analysis

Figures 5G,H illustrates the learning curves for mOSATS score and suturing time across the 10 training trials. Although both groups improved over time, the guidance group demonstrated earlier and greater gains in technical performance. The mOSATS score in the guidance group increased from 11.8 ± 2.6 at Trial 1 to 20.9 ± 2.6 at Trial 10 (78.0% improvement), compared with 11.8 ± 3.1 to 17.7 ± 3.3 in the control group (50.4% improvement). Similarly, suturing time decreased from 635.2 ± 77.2 to 310.4 ± 29.3 s in the guidance group, vs. 629.1 ± 80.4 to 382.5 ± 50.5 s in the control group. These descriptive trajectories suggest that visual trajectory guidance may be associated with greater short-term improvement in technical performance.Notably, Trials 1, 5, and 10 were conducted as independent assessment sessions without visual trajectory guidance, yet the guidance group continued to outperform the control group at these time points. This finding suggests that the benefit gained during guided practice was not limited to immediate cueing and may reflect short-term consolidation of guided practice effects. However, the present design does not establish definitive motor internalization or durable learning, which would require delayed retention testing. Collectively, these learning-curve data support the potential of AI-driven expert trajectory guidance to facilitate short-term improvement in novice renal wound suturing performance.

At completion of training, all 12 participants in the guidance group completed a system evaluation questionnaire. Ratings were favorable for clarity of the trajectory overlay, assistance in identifying the correct needle direction, and willingness to recommend incorporation of the system into renal suturing training curricula. The relatively lower score for progressive reduction in dependence on guidance suggested that, although the system was perceived as beneficial, novice trainees still preferred continuous visual assistance during task performance in the early phase of skill acquisition (Supplementary Table S8).

## 4 Discussion

### 4.1 Main contribution and significance

Renorrhaphy is a critical step in robot-assisted partial nephrectomy, and its quality directly affects intraoperative hemostasis, postoperative complications, and long-term renal function preservation (28). Despite rapid progress in surgical artificial intelligence, most current applications remain confined to recognition and assessment tasks, such as instrument detection, phase recognition, and postoperative skill evaluation, while studies that can be further translated into intraoperative guidance and skills training remain limited (29). Recent studies in the microvascular anastomosis domain have provided important precedents for AI-based surgical motion analysis. On et al. demonstrated that deep learning-based hand tracking can quantify expert microsurgical motion patterns, including economy and flow of motion, during simulated microvascular anastomosis, highlighting the potential of vision-based motion tracking for surgical training (30). Chen et al. further developed an LSTM-based model to evaluate procedural consistency in microvascular anastomosis by comparing predicted and actual suturing executions, providing an important example of AI-derived motion modeling for objective technical assessment (31). Building on these advances, we established an end-to-end framework for renal wound suturing that integrates multicenter expert trajectory data, a scene-aware Transformer, and prospective training validation. The trajectory-guided group significantly outperformed the control group in 6 of 8 performance measures, with large effect sizes, and these advantages persisted during unguided assessment sessions. Thus, our study extends AI-assisted surgical motion analysis from external hand-motion tracking and retrospective performance assessment to endoscopic video-based instrument trajectory prediction, intra-field expert trajectory visualization.

Related advances have also been reported in robotic urologic surgery and robot-assisted minimally invasive surgery training. De Backer et al. demonstrated real-time deep learning-based instrument delineation during augmented reality-guided robotic renal surgery, showing that computer vision can enhance intraoperative visualization in a renal surgical setting (32). Malpani et al. developed real-time virtual reality-based teaching cues for robot-assisted minimally invasive surgery training and showed in a randomized controlled trial that automated graphical guidance could improve surgical technique during needle passing (33). These studies support the broader potential of real-time visual augmentation and automated teaching in robotic surgery. Our work complements these efforts by focusing on expert trajectory prediction from standard endoscopic video and evaluating whether intra-field trajectory guidance can improve renal wound suturing skill acquisition in a prospective training study.

Although prior studies have shown accurate modeling of instrument motion using robotic kinematic data, such approaches still rely on platform-specific sensor interfaces and are therefore difficult to generalize across training environments (34, 35). In contrast, our framework relies only on standard endoscopic video and does not require platform-specific kinematic sensors. In principle, this may simplify deployment across different surgical platforms. However, because all data in the present study were acquired from a single robotic system (da Vinci Xi), generalizability to other robotic platforms and to other tissue types remains to be empirically validated. In our framework, the fine-tuned YOLOv13x detector served as the upstream perception module for instrument localization and visual feature extraction, whereas the Scene-Aware Transformer was designed as a compact trajectory prediction module operating on extracted coordinates and visual features. Therefore, the efficiency claim in this study refers to the trajectory prediction module rather than the entire annotation and perception pipeline. Compared with Transformer-based trajectory prediction baselines, the Scene-Aware branch added only 0.35 million parameters (13.1% of the prediction module) while improving ADE and FDE by 14.3% and 16.5%, respectively. Together, these results support the added value of visual scene context for surgical instrument motion prediction.

Pretrained tracking systems also warrant consideration. In the microvascular anastomosis literature, MediaPipe-based hand tracking and subsequent LSTM-based modeling has demonstrated the feasibility of using AI-derived motion data to assess procedural consistency (30, 31). However, the present task differs in that the target is not the surgeon's external hand motion but the robotic instrument clasper within a crowded endoscopic surgical field, where occlusion, specular reflection, tissue deformation, and instrument-tissue interaction are common. These differences make direct application of generic hand-tracking systems less suitable for our setting. Nevertheless, future work should systematically compare fine-tuned surgical detectors with general pretrained or foundation-model-based tracking approaches, including keypoint-based instrument models, to reduce annotation burden and improve cross-domain generalizability.

A major concern in AI-assisted procedural guidance is deskilling, whereby reliance on AI may impair independent performance. Recent multicenter observational data in colonoscopy suggested that prior exposure to AI-assisted adenoma detection was associated with worse unassisted detection performance after AI withdrawal, identifying AI exposure as an independent predictor of diminished unassisted detection performance (36). Our study directly addressed this concern by incorporating 3 unguided assessment sessions. The trajectory-guided group maintained superior performance during these unguided evaluations and outperformed the control group in 6 key metrics at final assessment, suggesting that the benefit of guidance was not limited to immediate cueing but may reflect internalization of expert motor patterns. Recent randomized trials have similarly shown that real-time AI feedback can achieve performance gains comparable to, or greater than, instructor-led teaching (37), and that combining AI support with expert instruction may reduce overreliance on any single feedback modality (38). Compared with these studies, our study extends AI-based training from generic real-time feedback to expert trajectory-based visual guidance in renal wound suturing, a reconstructive task with greater procedural specificity and clinical relevance.

AI-driven expert trajectory guidance may complement the traditional apprenticeship model of surgical training, particularly during early skill acquisition for complex reconstructive tasks. This direction is consistent with broader trends in minimally invasive therapy, where robotic platforms are increasingly moving toward procedure-specific guidance, image-guided navigation, and human-machine collaboration across specialties (39). Current training curricula, including the Fundamentals of Laparoscopic Surgery (40), emphasize generic technical skills and do not yet provide procedure-specific visual guidance for complex reconstructive tasks such as renal wound suturing. Evidence indicates that variable practice incorporating progressive difficulty adjustments promotes skill retention and transfer more effectively than fixed-pattern training (41). Building on this premise, future development of the system could incorporate adaptive fading, with gradual reduction of guidance intensity as proficiency improves, to further promote autonomous skill consolidation.

### 4.2 Limitations and future research directions

Several limitations should be acknowledged. First, this was a small pilot feasibility study that was not powered for definitive efficacy comparisons; therefore, the results require further validation in multicenter trials with formal sample size estimation (42). The task-specific mOSATS adaptation should also be interpreted as exploratory despite high inter-rater reliability. Second, training was performed on standardized *ex vivo* porcine kidneys, which do not fully reproduce the complexity of live RAPN, including bleeding, respiratory motion, tissue deformation, tumor variability, hilar control, ischemia pressure, and real operative stress. Long-term skill retention and transfer to the operating room were not assessed and require longitudinal follow-up in future studies (43). Third, although YOLO-based trajectory annotation was validated against independent manual annotation of the needle-grasping point, residual localization noise and a systematic geometric offset between the bounding-box center and the needle-grasping point remained. Future work could incorporate sub-pixel keypoint regression, marker-based ground truth, or direct robotic kinematic data if available to further reduce annotation noise and provide anatomically aligned references. Finally, the reported 32.7 ms inference latency was measured on a high-end laboratory workstation equipped with an NVIDIA RTX 5,090 GPU. Deployment on more typical operating-room computing systems may require model compression, hardware optimization, or edge-device acceleration, and real-time performance should be validated under realistic clinical video-routing conditions.

Despite these limitations, this prospective feasibility study provides evidence support for the AI-based visual guidance in surgical training. Future work should include multicenter randomized trials with stratification by institution and baseline experience, as well as evaluation in other complex reconstructive tasks such as vesicourethral anastomosis and hepatic parenchymal suturing.

## 5 Conclusion

In this pilot feasibility study, AI-based expert trajectory guidance derived from standard surgical video was feasible and was associated with improved short-term acquisition of robotic renal wound suturing skills in an *ex vivo* training model. These preliminary findings support further evaluation in larger multicenter randomized studies assessing long-term retention, transfer to clinical performance, safety-related outcomes, and safe integration into surgical training curricula.

## Data Availability

The raw data supporting the conclusions of this article will be made available by the authors, without undue reservation.
