# Supplementary material for: Artificial intelligence-based expert trajectory guidance in an ex vivo robot-assisted renal wound suturing training model
Source: Front Surg. 2026 Jul 2;13:1874149. doi: 10.3389/fsurg.2026.1874149 (PMC13373058; doi:10.3389/fsurg.2026.1874149)
Supplement: Supplementary file 1 [file Supplementaryfile1.docx]

***Supplementary Materials***

**Table of Supplementary Contents:**

- Supplementary Figure 1. Semantic Segmentation for RAPN Renorrhaphy
- Supplementary Figure 2. Validation of YOLO-Based Trajectory Annotation

Against Manual Annotation

- Supplementary Figure 3. Trajectory Characteristics of the Expert Suturing Dataset
- Supplementary Figure 4. Model Performance and Training Outcomes
- Supplementary Table 1. Patient and Procedural Characteristics
- Supplementary Table 2. Demographics of Participants
- Supplementary Table 3. Elements of Modified OSATS
- Supplementary Table 4. YOLOv13x Detection Performance on Test Set
- Supplementary Table 5. Quantitative Validation of YOLO-Based Trajectory Annotation Against Manual Annotation
- Supplementary Table 6. Quantitative Comparison of Trajectory Prediction Models
- Supplementary Table 7. Surgical Performance Between Guidance and Control Groups
- Supplementary Table 8. Participant-Reported Evaluation of Trajectory Guidance System
- Supplementary Methods 1. Evaluation for YOLO-Based Trajectory Annotation Against Manual Annotation
- Supplementary Methods 2. Scene-Aware Transformer Architecture and Training Details
- Supplementary Methods 3. Transfer Fine-Tuning for Ex Vivo Environment Adaptation

**
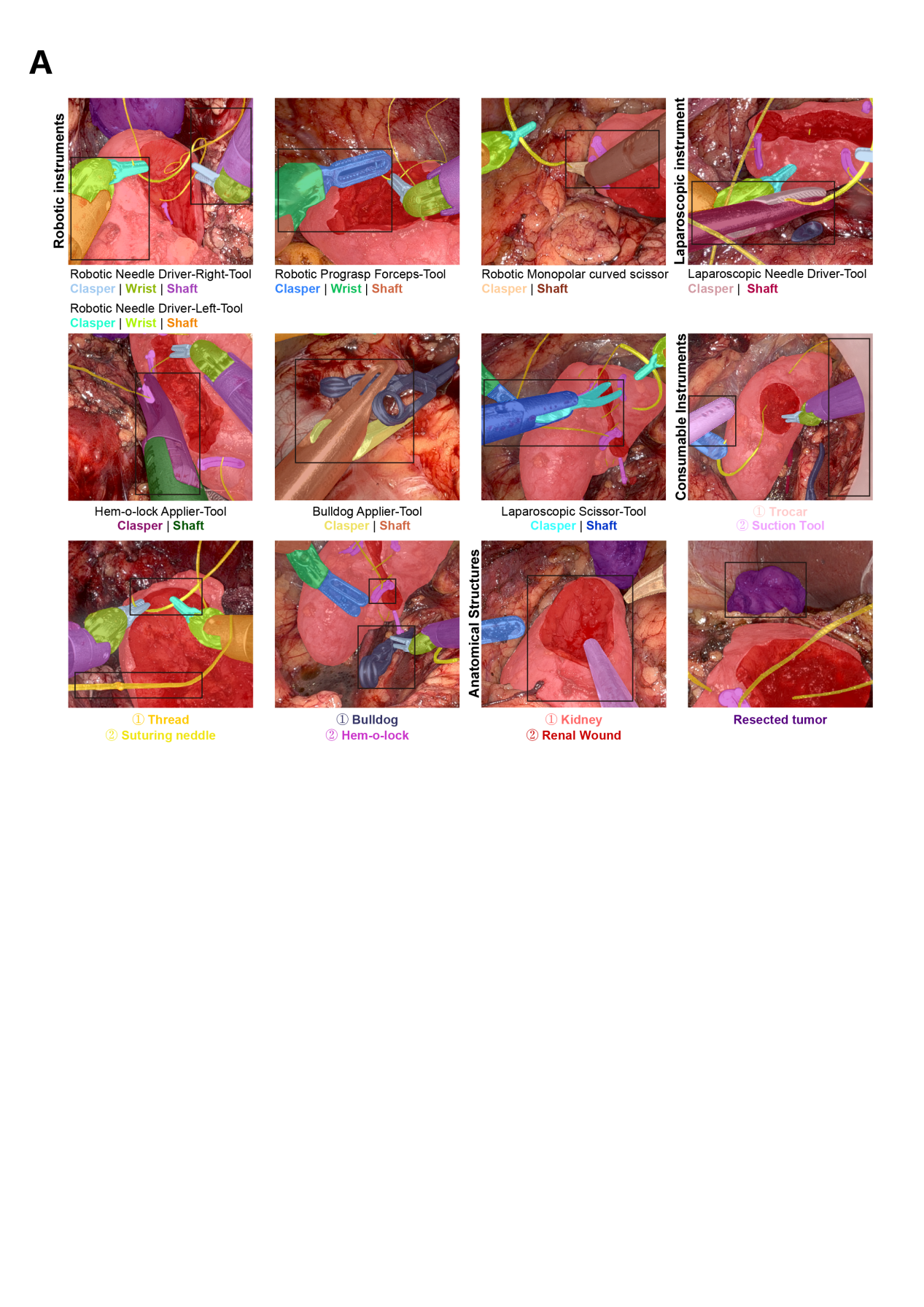
**

**Supplementary Figure 1.** Semantic segmentation for RAPN renorrhaphy. **(A)** Representative examples of semantic segmentation in renorrhaphy during RAPN, including robotic instruments (robotic needle drivers, Prograsp forceps, including their tool clasper, wrist, and shaft components), laparoscopic instruments (laparoscopic needle driver, Hem-o-lock applier, bulldog applier, laparoscopic scissor), consumable instruments (suction tool, suturing needle, suture thread, bulldog clamp, Hem-o-lock clip, trocar), and anatomical structures (renal wound, kidney parenchyma, resected tumor).


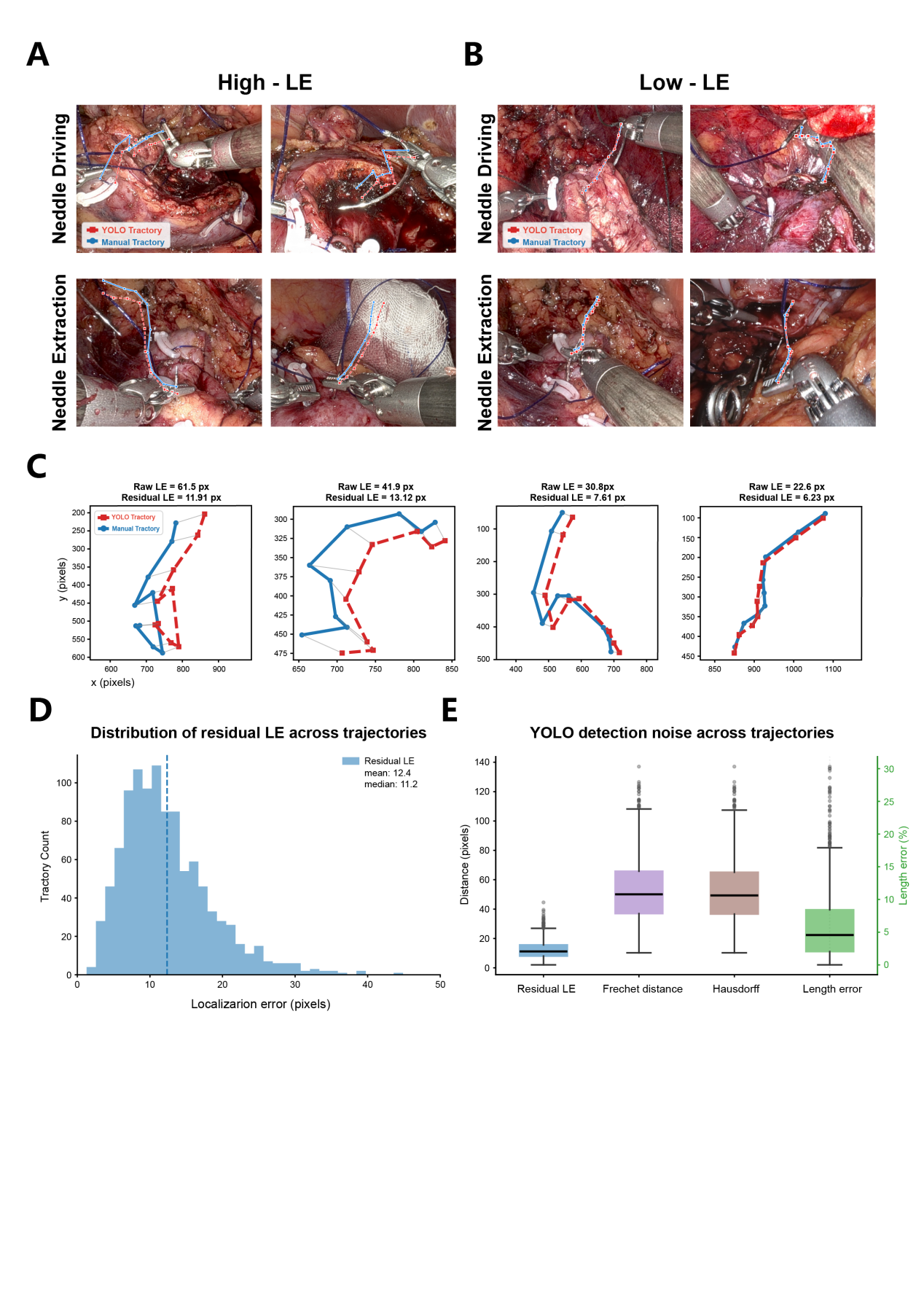


**Supplementary Figure 2.** Validation of YOLO-based trajectory annotation against manual annotation. **(A-B)** Representative frames overlaid with YOLO-derived (red dashed lines) and manually annotated (blue solid lines) trajectories, showing samples with high (A) and low (B) raw localization errors (Raw LE). Upper row: needle-driving; lower row: needle-extraction. **(C)** Coordinate-plane comparisons of four representative trajectories, arranged left to right by descending Raw LE. Grey lines connect paired keyframes. **(D)** Distribution of per-trajectory residual localization error (Residual LE). Dashed line indicates the mean. **(E)** Box plots of trajectory-level agreement metrics: Residual LE, discrete Fréchet distance, Hausdorff distance, and trajectory-length error. Raw LE = raw localization error; Residual LE = Raw LE after removing the per-trajectory systematic offset. Full metric definitions are provided in Supplementary Methods 1.


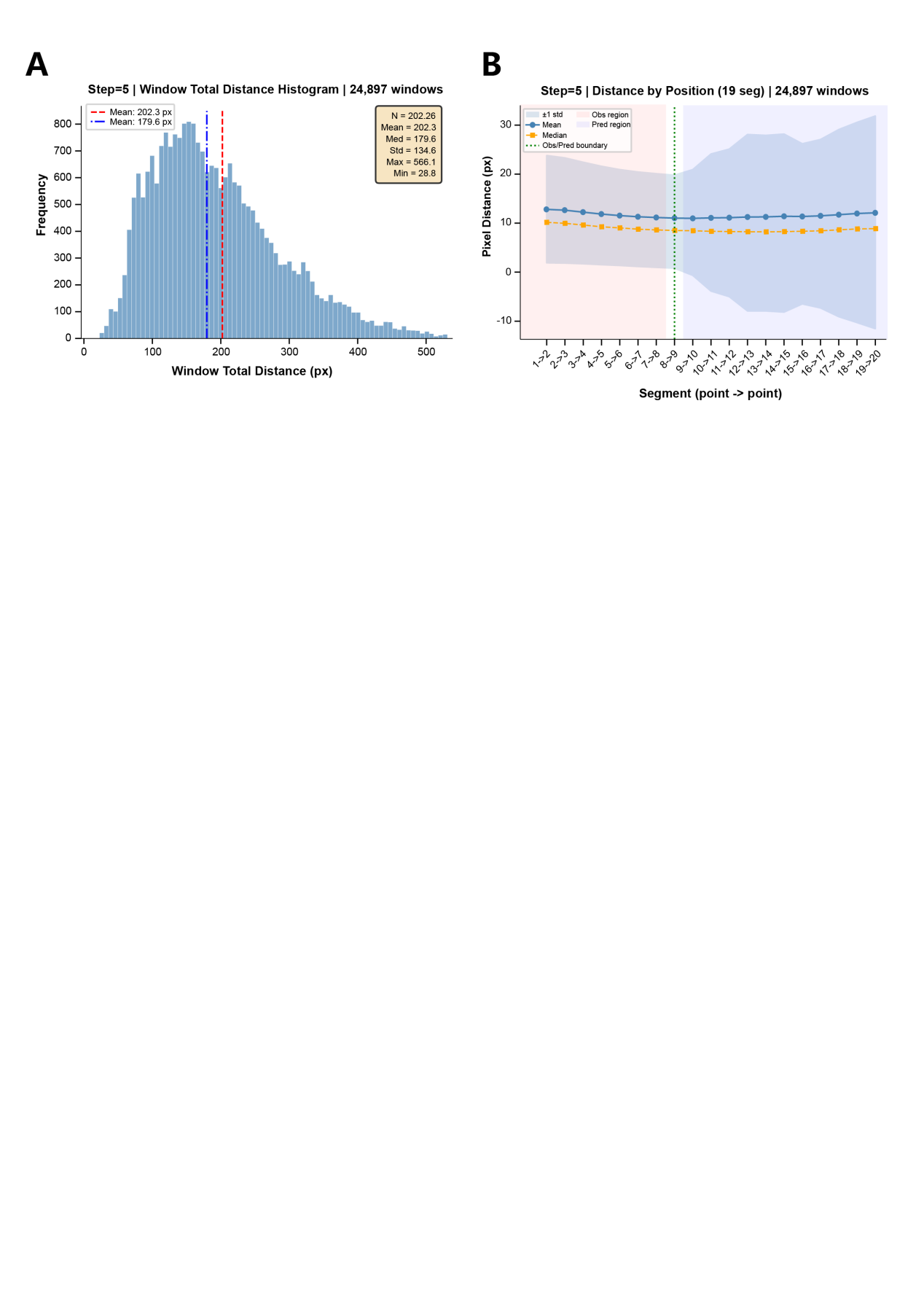
**Supplementary Figure 3.** Trajectory characteristics of the expert suturing dataset. **(A)** Histogram of total trajectory distance across all valid sliding-window samples (n = 24,897). **(B)** Distribution of point-to-point distance across all valid sliding-window samples.

**
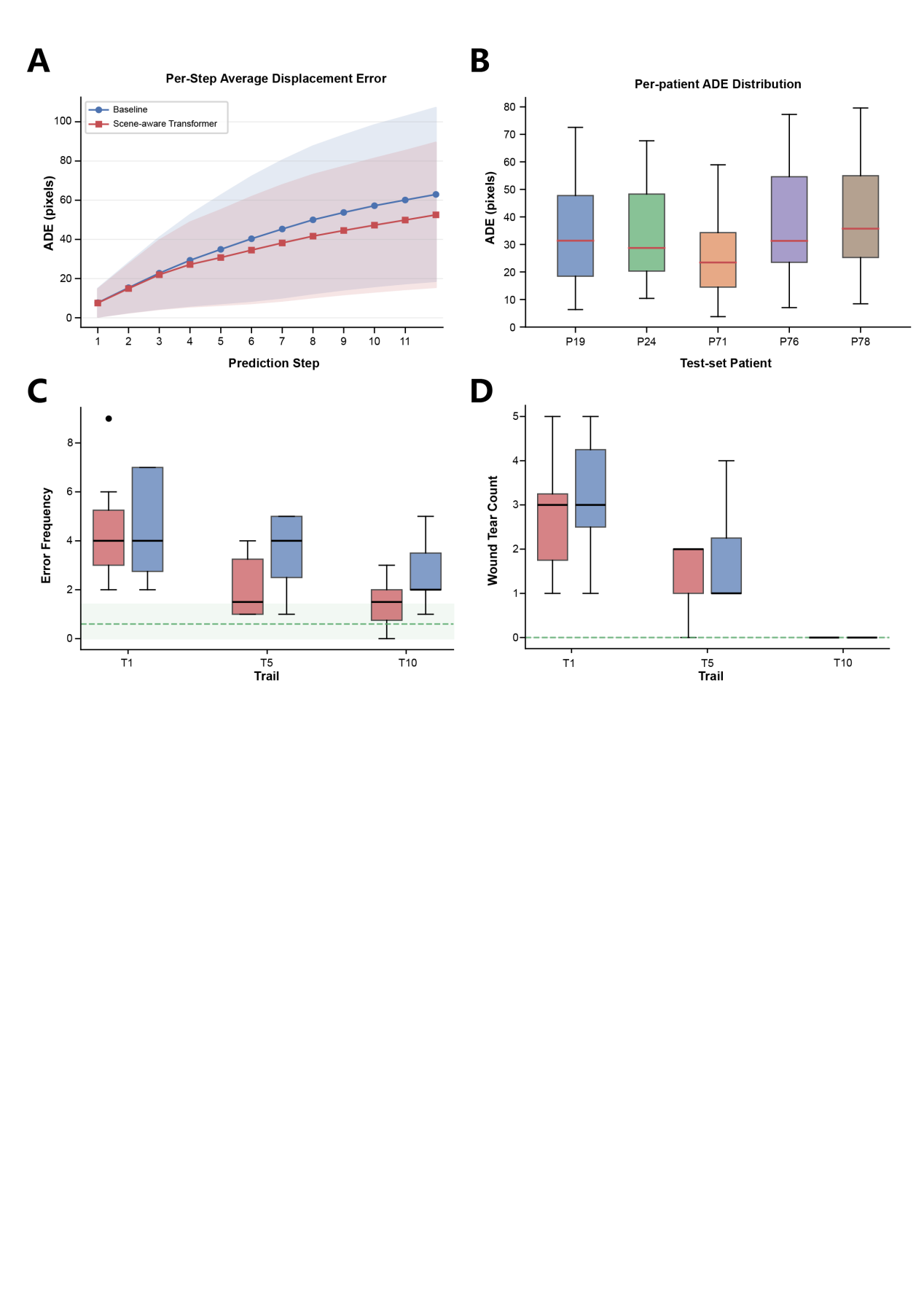
**

**Supplementary Figure 4.** Model performance and training outcomes. **(A)** Per-step ADE across the prediction horizon for the baseline model and the Scene-aware Transformer. **(B)** Per-patient ADE distribution in the independent test set. **(C-D)** Error frequency and wound tear count in the guidance and control groups at baseline, midpoint, and final assessment, with expert baseline shown for reference.

**Supplementary Table 1. Patient and procedural characteristics of the surgical video dataset.**

| **Characteristic** | **Training set (n=40)** | **Validation set (n=5)** | **Test set (n=5)** | **Total (n=50)** |
| --- | --- | --- | --- | --- |
| **Sex** |  |  |  |  |
| Male | 26 (65.0%) | 4 (80.0%) | 2 (20.0%) | 32 (64.0%) |
| Female | 14 (35.0%) | 1 (20.0%) | 3 (60.0%) | 18 (34.0%) |
| **Age (years)** | 53 (28-76) | 55 (40-56) | 48 (44-54) | 53 (28-76) |
| **Laterality** |  |  |  |  |
| Left | 23 (57.5%) | 3 (60.0%) | 4 (80.0%) | 30 (63.8%) |
| Right | 17 (42.5%) | 2 (40.0%) | 1 (20.0%) | 20 (36.2%) |
| **Approach** |  |  |  |  |
| Transabdominal | 14 (35.0%) | 1 (20.0%) | 2 (40.0%) | 17 (29.8%) |
| Retroperitoneal | 26 (65.0%) | 4 (80.0%) | 3 (60.0%) | 33 (70.2%) |
| **Wound size** |  |  |  |  |
| Large | 17 (42.5%) | 2 (40.0%) | 2 (40.0%) | 21 (42.6%) |
| Medium | 21 (52.5%) | 3 (60.0%) | 3 (60.0%) | 27 (53.2%) |
| Small | 2 (5.0%) | 0 (0.0%) | 0 (0.0%) | 2 (4.3%) |

Data are presented as median (range) or n (*%*).

**Supplementary Table 2. Demographics of participants.**

| **Characteristic** | **Guidance Group (N=12)** | **Control Group (N=12)** | **P** |
| --- | --- | --- | --- |
| **Sex** |  |  | 1.00 |
| Male | 10 (83.3%) | 11 (91.7%) |  |
| Female | 2 (16.7%) | 1 (8.3%) |  |
| **Age (years)** | 26 (24–28) | 25 (24–27) | 0.874 |
| **Postgraduate year** |  |  | 1.00 |
| PGY 1 | 5 (41.7%) | 6 (50.0%) |  |
| PGY 2 | 7 (58.3%) | 6 (50.0%) |  |
| **Prior laparoscopic experience (hours)** | 4.8 (2.0–8.0) | 5.0 (2.5–8.5) | 0.928 |
| **Prior suture training** |  |  | 1.00 |
| Yes | 6 (50.0%) | 7 (58.3%) |  |
| No | 6 (50.0%) | 5(41.7%) |  |

Data are presented as median (range) or n (*%*). P values were calculated using the Mann–Whitney U test for continuous variables and Fisher's exact test for categorical variables.

**Supplementary Table 3. Elements of modified objective structured assessment of technical skills (mOSATS)**

| **Elements** | **Rating scale** |
| --- | --- |
| Respect of tissue | 1 - Frequently used unnecessary force on tissue;  3 - Careful tissue handling but occasionally caused inadvertent damage;  5 - Consistent appropriate tissue handling |
| Suture/needle handling | 1 - Awkward and unsure with repeated entanglement and poor knot tying;  3 - Majority of knots placed correctly with appropriate tension;  5 - Excellent suture control |
| Time and motion | 1 - Made unnecessary moves;  3 - Efficient time/motion but some unnecessary moves;  5 - Clear economy of movement and maximum efficiency |
| Flow of operation | 1 - Frequently interrupted flow to discuss the next move;  3 - Demonstrated some forward planning and reasonable procedure progression;  5 - Obviously planned course of operation with efficient transitions between moves |
| Overall performance | 1 - Very poor;  3 - Competent;  5 - Clearly superior |
| Quality of final product | 1 - Very poor;  3 - Competent;  5 - Clearly superior |

Each domain is scored on a 5-point Likert scale (1–5), with anchor descriptors at 1, 3, and 5. Scores of 2 and 4 represent intermediate performance between the adjacent anchors. The total mOSATS score is the sum of all six domains (range: 6–30).

**Supplementary Table 4. Performance of YOLOv13 for surgical instrument components and anatomical structures on test set.**

| **Class Name** | **Precision** | **Recall** | **mAP50** | **mAP75** | **mAP50-95** | **Ground-truth Count** |
| --- | --- | --- | --- | --- | --- | --- |
| **Robotic instruments** |  |  |  |  |  |  |
| *Robotic Needle Driver-Right-Tool Clasper | 0.968 | 0.965 | 0.985 | 0.964 | 0.884 | 1223 |
| Robotic Needle Driver-Right-Tool Wrist | 0.973 | 0.972 | 0.986 | 0.954 | 0.872 | 1168 |
| Robotic Needle Driver-Right-Tool Shaft | 0.918 | 0.959 | 0.972 | 0.956 | 0.922 | 1073 |
| *Robotic Needle Driver-Left-Tool Clasper | 0.945 | 0.943 | 0.968 | 0.901 | 0.817 | 1861 |
| Robotic Needle Driver-Left-Tool Wrist | 0.978 | 0.959 | 0.984 | 0.948 | 0.869 | 1930 |
| Robotic Needle Driver-Left-Tool Shaft | 0.976 | 0.955 | 0.985 | 0.969 | 0.937 | 1785 |
| *Robotic Prograsp Forceps-Tool Clasper | 0.931 | 0.926 | 0.937 | 0.921 | 0.865 | 934 |
| Robotic Prograsp Forceps-Tool Wrist | 0.968 | 0.934 | 0.969 | 0.915 | 0.874 | 759 |
| Robotic Prograsp Forceps-Tool Shaft | 0.904 | 0.776 | 0.867 | 0.839 | 0.792 | 407 |
| **Laparoscopic instrument** |  |  |  |  |  |  |
| Laparoscopic Needle Driver-Tool Clasper | 0.826 | 0.410 | 0.625 | 0.468 | 0.434 | 93 |
| Laparoscopic Needle Driver-Tool Shaft | 0.878 | 0.810 | 0.851 | 0.803 | 0.709 | 169 |
| Hem-o-lock Applier-Tool Clasper | 0.930 | 0.859 | 0.919 | 0.865 | 0.824 | 135 |
| Hem-o-lock Applier-Tool Shaft | 0.812 | 0.666 | 0.758 | 0.710 | 0.647 | 45 |
| Bulldog Applier-Tool Clasper | 0.351 | 0.294 | 0.253 | 0.253 | 0.250 | 17 |
| Bulldog Applier-Tool Shaft | 0.245 | 0.454 | 0.242 | 0.242 | 0.240 | 11 |
| Laparoscopic Scissor-Tool Clasper | 0.795 | 0.708 | 0.767 | 0.583 | 0.497 | 11 |
| Laparoscopic Scissor-Tool Shaft | 0.896 | 0.777 | 0.892 | 0.892 | 0.786 | 9 |
| **Consumable Instruments** |  |  |  |  |  |  |
| Suction Tool | 0.952 | 0.917 | 0.973 | 0.952 | 0.946 | 426 |
| Suturing Needle | 0.866 | 0.891 | 0.920 | 0.868 | 0.804 | 2036 |
| Thread | 0.881 | 0.852 | 0.919 | 0.830 | 0.793 | 4591 |
| Bulldog | 0.960 | 0.826 | 0.941 | 0.817 | 0.767 | 1202 |
| Hem-o-lock | 0.936 | 0.803 | 0.921 | 0.852 | 0.782 | 3837 |
| Trocar | 0.941 | 0.942 | 0.958 | 0.958 | 0.928 | 157 |
| **Anatomical Structures** |  |  |  |  |  |  |
| Renal Wound | 0.885 | 0.750 | 0.867 | 0.745 | 0.690 | 5236 |
| Kidney | 0.841 | 0.674 | 0.797 | 0.604 | 0.584 | 8009 |
| Resected Tumor | 0.892 | 0.309 | 0.512 | 0.363 | 0.330 | 917 |

Precision: the ratio of true positive detections to the total number of predicted bounding boxes, reflecting the model's ability to avoid false positives; Recall: the ratio of true positive detections to the total number of ground-truth instances, measuring the model's ability to detect all relevant objects; mAP50/75: mean Average Precision at an Intersection over Union (IoU) threshold of 0.50 /0.75; mAP50-95: mean Average Precision averaged over ten IoU thresholds from 0.50 to 0.95 (step 0.05); Ground-truth Count: the total number of annotated instances for each class in the test set. *Key surgical instruments used during suturing

**Supplementary Table 5. Quantitative validation of YOLO-based trajectory annotation against manual annotation.**

| **Metric** | **Needle-driving**  **(n = 500)** | **Needle-extraction**  **(n = 500)** | **Overall**  **(n = 1,000)** |
| --- | --- | --- | --- |
| Raw localization error (px) | 35.88 ± 18.40 | 41.50 ± 22.63 | 38.80 ± 20.89 |
| Residual localizarion error (px) | 10.92 ± 8.13 | 13.69 ± 10.21 | 12.36 ± 9.37 |
| Trajectory-length error (%) | 5.40 ± 4.93 | 6.84 ± 6.02 | 6.15 ± 5.57 |
| Discrete Fréchet distance (px) | 48.77 ± 19.11 | 56.75 ± 23.39 | 52.91 ± 21.81 |
| Hausdorff distance (px) | 48.02 ± 18.72 | 56.40 ± 23.34 | 52.37 ± 21.65 |

Values are mean ± SD. Raw LE, raw localization error; Residual LE, raw LE after removing the per-trajectory systematic offset between the bounding-box center and the needle-grasping point; discrete Fréchet distance and Hausdorff distance, trajectory-level metrics quantifying the overall shape similarity between paired YOLO-derived and manual trajectories; trajectory-length error, the absolute difference in cumulative path length between the YOLO-derived and manual trajectories, normalized by the manual trajectory length. Full metric definitions are provided in Supplementary Methods 1.

**Supplementary Table 6. Quantitative comparison of trajectory prediction models**

| **Method** | **Prediction Performance** | | | | |
| --- | --- | --- | --- | --- | --- |
|  | **ADE (↓)** | **FDE (↓)** | **FD (↓)** | **Params (×10^4^)** | **FLOPs (M) (↓)** |
| **MemoNet** | 74.32 ± 46.62 | 88.13 ± 64.17 | 94.17 ± 61.61 | 558.98 | 21.83 |
| **Trajectron++** | 71.34 ± 42.81 | 86.41 ± 42.11 | 91.56 ± 41.34 | **21.47** | **4.17** |
| **MID** | 66.52 ± 38.76 | 79.27 ± 46.32 | 83.08 ± 43.70 | 1,220.35 | 16,764 |
| **PPT** | 46.24 ± 32.58 | 70.59 ± 40.98 | 76.55 ± 36.41 | 256.19 | 38.17 |
| **Singulartrajectory** | 42.89 ± 27.01 | 65.94 ± 37.01 | 69.33 ± 34.49 | 336.49 | 320.87 |
| **Baseline Model (Ours)** | 39.97 ± 24.31 | 62.66 ± 37.65 | 65.27 ± 36.75 | 232.73 | 46.20 |
| **+ Element-wise addition** | 38.94 ± 23.76 | 61.52 ± 36.88 | 63.81 ± 35.92 | 254.96 | 47.98 |
| **+ Concatenation** | 38.12 ± 23.21 | 60.34 ± 36.15 | 62.45 ± 35.08 | 262.31 | 49.12 |
| **+ Gated fusion** | 36.95 ± 22.34 | 56.83 ± 34.91 | 59.12 ± 34.06 | 259.78 | 50.24 |
| **SAT (Ours, + Cross-attention) Seed128** | 36.32 ± 20.71 | 56.08 ± 32.48 | 60.19 ± 33.91 | 267.40 | 52.82 |
| **SAT (Ours, + Cross-attention) Seed357** | **34.25 ± 19.32** | **52.54 ± 31.71** | **53.99 ± 30.61** | 267.40 | 52.82 |
| **SAT (Ours, + Cross-attention) Seed492** | 36.07 ± 21.58 | 55.77 ± 32.93 | 58.81 ± 34.24 | 267.40 | 52.82 |

Bold values indicate the best performance. ↓ denotes lower is better. ADE, average displacement error, defined as the mean Euclidean distance between predicted and ground-truth positions across all predicted time steps; FDE, final displacement error, defined as the Euclidean distance at the last predicted time step; FD, Discrete Fréchet Distance, evaluating global shape similarity between the predicted and ground-truth trajectories; Params, number of trainable parameters in millions; FLOPs, floating-point operations for a single forward pass (encoder + scene encoding + one decoder step), in millions (M). Baseline (Ours) denotes the trajectory-only Transformer without the scene-aware branch. '+ Concatenation', '+ Element-wise addition', and '+ Gated fusion' denote ablation variants that integrate the scene-aware branch into the Baseline using the corresponding fusion strategy, while keeping all other components identical. SAT (Ours) denotes the proposed Scene-aware Transformer with cross-attention fusion, representing the optimal fusion strategy among those compared. For the best-performing model (Seed357), bootstrap 95% confidence intervals were 33.47–35.03 pixels for ADE and 51.26–53.82 pixels for FDE. All models were evaluated under the same observation length (Obs = 8 frames) and prediction horizon (Pred = 12 frames) on the identical held-out test set.

**Supplementary Table 7. Surgical performance between guidance and control groups.**

| **Variable** | **Trial 1 (Baseline)** | | **Trial 5** | | **Trial 10** | | **Expert** | **P^a^** | **P^b^** | **P^c^** |
| --- | --- | --- | --- | --- | --- | --- | --- | --- | --- | --- |
|  | **Guidance** | **Control** | **Guidance** | **Control** | **Guidance** | **Control** | **Baseline** |  |  |  |
| **Suturing Time (Sec)** | 635.2 ± 77.2  (485.9 - 733.0) | 629.1 ± 80.4  (475.2 - 734.7) | 369.9 ± 69.5 (290.7 - 504.2) | 459.4 ± 51.2 (378.5 - 544.7) | 310.4 ± 29.3 (257.1 - 345.7) | 382.5 ± 50.5  (326.1 - 495.3) | 225.6 ± 29.7  (186.0 - 270.3) | 0.885 | 0.010* | <0.001** |
| **Time per Stitch (Sec/stitch)** | 103.0 ± 18.1  (73.5 - 123.2) | 100.2 ± 12.7 (82.0 - 124.7) | 60.6 ± 7.5 (48.4 - 72.0) | 76.4 ± 14.2 (60.0 - 97.0) | 49.2 ± 7.6 (38.7 - 64.2) | 61.7 ± 9.9 (45.4 - 84.0) | 38.5 ± 5.2 (31.8 - 45.7) | 0.707 | 0.023* | 0.009** |
| **Instrument-Needle Interaction** | 28.8± 5.4  (19 - 38) | 28.2 ± 4.5  (20 - 36) | 21.9 ± 3.2  (17 - 27) | 24.3 ± 3.6  (20 - 32) | 18.4 ± 2.3  (15 - 22) | 22.3 ± 3.3  (18 - 28) | 14.8 ± 1.8  (12 - 17) | 0.839 | 0.171 | 0.013* |
| **Error Frequency** | 4.5 ± 2.1  (1 - 9) | 4.5 ± 2.1  (2 - 7) | 2.4 ± 1.3  (1 - 5) | 3.4 ± 1.6  (1 - 5) | 1.6 ± 1.0  (0 - 3) | 2.8 ± 1.5  (1 - 5) | 0.7 ± 0.7  (0 - 2) | 0.997 | 0.131 | 0.061 |
| **Maximum Gap Length (mm)** | 9.8 ± 2.9  (5.3 - 14.7) | 9.5 ± 3.4  (4.5 - 14.6) | 5.1 ± 1.7  (1.9 - 7.6) | 6.4 ± 2.5  (2.5 - 10.5) | 3.5 ± 1.0  (1.4 - 5.1) | 5.3 ± 1.7  (2.5 - 8.3) | 1.1 ± 0.3  (0.7 - 1.8) | 0.795 | 0.225 | 0.026* |
| **Total Gap Length (mm)** | 16.1 ± 5.3  (8.8 - 25.8) | 16.7 ± 5.4  (7.8 - 26.6) | 9.8 ± 2.0  (6.4 - 12.4) | 12.1 ± 4.4  (5.9 - 21.1) | 5.1 ± 1.9  (2.9 - 9.0) | 8.3 ± 3.8  (3.7 - 14.2) | 2.8 ± 1.1  (0.6 - 3.9) | 0.798 | 0.263 | 0.032* |
| **Wound Tear Count** | 2.8 ± 1.4  (1 - 5) | 3.2 ± 1.5  (1 - 5) | 1.5 ± 0.8  (0 - 2) | 1.8 ± 1.1  (0 - 4) | 0.0 ± 0.0  (0 - 0) | 0.0 ± 0.0  (0 - 0) | 0.0 ± 0.0  (0 - 0) | 0.593 | 0.829 | 1 |
| **mOSATS Score** | 11.8 ± 2.6  (7 - 17) | 11.8 ± 3.1  (8 - 17) | 18.4 ± 2.2  (15 - 22) | 15.6 ± 2.9  (10 - 20) | 20.9 ± 2.6  (17 - 25) | 17.7 ± 3.3  (12 - 23) | 26.5 ± 2.4  (23 - 30) | 0.816 | 0.027* | 0.023* |

Values are presented as mean ± standard deviation (range). P^a^, P^b^, and P^c^ indicate between-group comparisons at Trial 1, Trial 5, and Trial 10, respectively, performed using the Mann-Whitney U test. Expert baseline values are shown for reference. mOSATS, modified Objective Structured Assessment of Technical Skills. *P < 0.05, **P < 0.01, ***P < 0.001. Cohen's d effect sizes for significant between-group differences at Trial 10 were: suturing time, 1.75; time per stitch, 1.42; instrument-needle interaction count, 1.39; mOSATS score, 1.09; maximum gap length, 1.25; and total gap length, 1.05 (all indicating large effects, d > 0.8). Rank-biserial correlations for significant Trial 10 between-group differences were: suturing time, -0.83; time per stitch, -0.64; instrument-needle interaction count, -0.60; maximum gap length, -0.54; total gap length, -0.54; and mOSATS score, 0.56. Negative values indicate lower values in the guidance group, whereas positive values indicate higher values in the guidance group. After Benjamini-Hochberg false-discovery-rate correction across the eight outcomes within each assessment time point, no Trial 5 comparison remained significant at q < 0.05, whereas all six significant Trial 10 outcomes remained significant (q = 0.0047–0.0350).

**Supplementary Table 8. Participant- reported evaluation of the trajectory guidance system in the guidance group**

| **Domain** | **Item** | **Guidance group (n = 12)** |
| --- | --- | --- |
| **Visual presentation** | Trajectory guidance lines were clearly visible in the surgical field | 4.58 ± 0.49 |
| **Visual presentation** | Trajectory guidance lines did not interfere with the normal operative view | 4.42 ± 0.49 |
| **Guidance effectiveness** | Trajectory guidance helped me understand the correct needle insertion direction and angle | 4.50 ± 0.50 |
| **Guidance effectiveness** | Trajectory guidance helped me control needle insertion depth and force | 4.25 ± 0.43 |
| **Guidance effectiveness** | Trajectory guidance helped reduce unnecessary repeated attempts | 4.33 ± 0.75 |
| **Learning effect** | After repeated training, my dependence on trajectory guidance gradually decreased | 3.75 ± 0.83 |
| **Learning effect** | Use of the system improved my confidence in renal wound suturing | 4.50 ± 0.76 |
| **Comparison with conventional training** | The system was superior to conventional observational video-based learning | 4.25 ± 0.72 |
| **Comparison with conventional training** | The system was superior to learning based solely on expert verbal instruction | 4.17 ± 0.80 |
| **Overall evaluation** | I would recommend incorporating this system into renal suturing training curricula | 4.58 ± 0.49 |
| **Overall evaluation** | Overall satisfaction with the system | 4.50 ± 0.50 |

Data are presented as mean ± SD. Items were rated on a 5-point Likert scale (1 = strongly disagree, 2 = disagree, 3 = neutral, 4 = agree, and 5 = strongly agree).

**Supplementary Methods 1. Evaluation for YOLO-based trajectory annotation against manual annotation**

**Evaluation Metrics**

Raw localization error (Raw LE), defined as the per-frame Euclidean distance between the YOLO-derived bounding-box center and the manually annotated needle-grasping point:

$$\text{Raw }\text{LE}_{i}=\sqrt{\left( x_{i}^{YOLO}-x_{i}^{manual} \right)^{2}+\left( y_{i}^{YOLO}-y_{i}^{manual} \right)^{2}}$$

Residual localization error (Residual LE), defined as the Euclidean distance after removing the per-trajectory systematic offset, isolating the random YOLO detection noise:

$$\text{Residual }\text{LE}_{i}=\sqrt{\left( x_{i}^{YOLO}-x_{i}^{manual}-\Delta x \right)^{2}+\left( y_{i}^{YOLO}-y_{i}^{manual}-\Delta y \right)^{2}}$$

The systematic offset magnitude (bias magnitude) summarizes the constant geometric difference between the bounding-box center and the needle-grasping point:

$$\text{Bias}=\sqrt{\Delta x^{2}+\Delta y^{2}}$$

Discrete Fréchet Distance (FD), evaluating the global shape similarity between the manually annotated trajectory A and the YOLO-derived trajectory B:

$$F\left( A,B \right)=ca\left( \left| A \right|,\left| B \right| \right)$$

$$ca\left( i,j \right)=\max\{\min\{ca\left( i-1,j \right),ca\left( i-1,j-1 \right),ca\left( i,j-1 \right)\},\left\| a_{i}-b_{j} \right\|\}$$

Hausdorff distance, evaluating the worst-case point-to-set deviation between the manually annotated trajectory A and the YOLO-derived trajectory B:

$$H\left( A,B \right)=\max\left\{ \sup_{a\in A} \inf_{b\in B} \left\| a-b \right\|, \sup_{b\in B} \inf_{a\in A} \left\| a-b \right\| \right\}$$

Trajectory-length error, defined as the absolute difference between the cumulative path lengths of the YOLO-derived and manually annotated trajectories, normalized by the manual trajectory length (a translation-invariant measure of shape consistency):

$$\text{Length error}=\frac{\left| L_{YOLO}-L_{manual} \right|}{L_{manual}}$$

**Supplementary Methods 2. Scene-Aware Transformer architecture and training details**

**Problem Formulation**

The instrument trajectory prediction task is formulated as follows. Given a sequence of $T_{\mathrm{obs}} = 8$ observed two-dimensional positions $\boldsymbol{P}^{\mathrm{obs}} = \{\left( X_{1},Y_{1} \right), \left( X_{2},Y_{2} \right), \ldots, \left( X_{8},Y_{8} \right)\}$ of the target end-effector clasper on the image plane, along with the corresponding $T_{\mathrm{obs}}$ video frames, the objective is to predict the subsequent $T_{\mathrm{pred}} = 12$ future positions ${\hat{\boldsymbol{P}}}^{\mathrm{pred}}=\{(\hat{X}_{9},\hat{Y}_{9}),...,((\hat{X}_{20},\hat{Y}_{20})\}$. Rather than directly regressing absolute coordinates, the model operates on velocity vectors (i.e., frame-to-frame displacements):

$$\boldsymbol{V}_{t} = \left( X_{t+1} - X_{t}, Y_{t+1} - Y_{t} \right), t = 1, \ldots, T_{\mathrm{obs}} - 1$$

yielding an input sequence of $T_{\mathrm{obs}} - 1 = 7$ velocity vectors. All velocity vectors are normalized to zero mean and unit variance using statistics computed from the training set. Predicted velocities are denormalized and cumulatively summed to recover absolute positions:

$${\hat{\boldsymbol{P}}}_{T_{\mathrm{obs}+k}}=\boldsymbol{P}_{T_{\mathrm{obs}}}+\sum_{i=1}^{k} ({\hat{\boldsymbol{V}}}_{i} \cdot\sigma+\mu), k = 1, \ldots, T_{\mathrm{pred}}$$

where $\mu$ and $\sigma$ denote the training-set velocity mean and standard deviation, respectively.

**Overall Architecture**

The proposed Scene-Aware Trajectory Transformer adopts a dual-branch encoder–decoder architecture, as illustrated in Figure 3. The trajectory encoding branch captures the temporal dynamics of the instrument motion from the velocity sequence, while the scene-aware branch extracts visual context from the corresponding video frames through a pre-trained object detection backbone. The two branches are fused via a cross-attention mechanism before being fed into an autoregressive decoder that generates future trajectory predictions. The overall architecture is described in detail below.

**Scene Feature Extraction**

To provide the model with visual scene context, frame-level features are extracted from each of the $T_{\mathrm{obs}} = 8$ observed video frames using the backbone of a fine-tuned YOLOv13x object detection model. Specifically, each frame is first cropped to remove non-informative border regions (yielding a 1342×1004 pixel effective field of view) and resized to 640×640 pixels. The image is then passed through the YOLOv13x backbone, and the output feature map is extracted.

A global average pooling operation is applied to the output feature map to produce a compact 768-dimensional feature vector per frame. This process yields a scene feature sequence

$$\boldsymbol{S}= \{s_{1}, s_{2}, \ldots, s_{8}\} \in{\mathbb{\mathbb{R}}}^{8\times768}$$

that encodes the spatial layout of instruments, surgical accessories, and anatomical structures across the observation horizon. All scene features are extracted offline and cached as NumPy arrays to decouple the computational cost of the detection backbone from the trajectory prediction training.

**Trajectory Encoding Branch**

The trajectory encoding branch transforms the observed velocity sequence into a high-dimensional trajectory representation. The input velocity vectors $\boldsymbol{V}_{1:7} \in{\mathbb{\mathbb{R}}}^{7\times2}$ are first projected into the model's latent space via a linear embedding layer scaled by $\sqrt{d_{\mathrm{model}}}$ :

$$\boldsymbol{e}_{t}^{\mathrm{traj}}=Linear(\boldsymbol{V}_{t})\cdot\sqrt{d_{\mathrm{model}}}$$

Sinusoidal positional encodings are then added to preserve temporal ordering. The resulting sequence is processed by a Transformer encoder consisting of $N = 4$ layers, each comprising multi-head self-attention with $h = 4$ heads and a position-wise feed-forward network with an inner dimension of $d_{\mathrm{ff}} = 256$. All encoder layers adopt the Pre-LayerNorm (Pre-LN) configuration for improved training stability. The output of the trajectory encoder is denoted as the trajectory memory $\boldsymbol{M}_{\mathrm{traj}} \in{\mathbb{\mathbb{R}}}^{d_{\mathrm{model}}}$.

**Scene-Aware Transformer Encoder**

The scene-aware branch processes the extracted visual features to capture how the surgical scene evolves over the observation window, reflecting instrument movement trends, tissue exposure changes, and spatial rearrangements of surgical accessories. The scene feature sequence $\boldsymbol{S} \in{\mathbb{\mathbb{R}}}^{8\times768}$  is first projected into the shared latent space through a linear layer followed by ReLU activation and dropout:

$$\boldsymbol{e}_{t}^{\mathrm{scene}}=Dropout\left( \mathrm{ReLU}\left( \mathrm{Linear}\left( \boldsymbol{S}_{t} \right) \right) \right)$$

After adding sinusoidal positional encodings, the sequence is passed through a dedicated Scene-Aware Transformer encoder comprising $\boldsymbol{N}_{\mathrm{scene}} = 2$ Pre-LN layers with $h_{\mathrm{scene}} = 8$ attention heads and a feed-forward dimension of $d_{\mathrm{ff}}^{\mathrm{scene}}=1024$. Through temporal self-attention, each frame's representation is contextualized by all other frames in the observation window, producing a temporally enriched scene memory $\boldsymbol{M}_{\mathrm{scene}} \in{\mathbb{\mathbb{R}}}^{8\times d_{\mathrm{model}}}$.

**Cross-Attention Fusion and Autoregressive Decoding**

The fused memory $\boldsymbol{M}_{\mathrm{fused}}$ is passed to a Transformer decoder that autoregressively generates the future velocity sequence. The decoder consists of $N = 6$ Pre-LN layers with the same configuration as the trajectory encoder ($h = 8$, $d_{\mathrm{ff}} =256$). Each decoder layer performs causal self-attention over the previously generated tokens (enforced by a causal mask) and cross-attention over the fused encoder memory.

At inference time, decoding is initiated with a start-of-sequence token $t_{0} = [0, 0, 1]$, where the third dimension serves as a binary flag distinguishing the start token from velocity predictions. At each step $k = 1, \ldots, T_{\mathrm{pred}}$ the decoder produces a three-dimensional output [$\hat{V}_{x}$*,*$\hat{V}_{y}$*,c*], of which only the first two dimensions are used as the predicted velocity. The output is appended to the decoder input sequence, and the process repeats for 12 steps. A linear generator layer projects the decoder output from $d_{\mathrm{model}}$ dimensions to the 3-dimensional output space.

**Training Objective**

The model is trained by minimizing a composite loss function. The primary term computes the mean pairwise Euclidean distance between the predicted and ground-truth normalized velocity vectors across all prediction time steps:

$$L_{\mathrm{traj}} = \frac{1}{B \cdot T_{\mathrm{pred}}}\sum_{b=1}^{B} \sum_{k=1}^{T_{\mathrm{pred}}} \left\| \hat{V}_{b,k}-V_{b,k}^{*} \right\|_{2}$$

An auxiliary regularization term encourages the third output dimension (the start-of-sequence flag channel) to approach zero during prediction:

$$L_{\mathrm{aux}}=\frac{1}{B\cdot T_{\mathrm{pred}}}\sum_{b=1}^{B} \sum_{k=1}^{T_{pred}} {|C}_{b,k}|$$

The total training loss is:

$${L=L}_{\mathrm{traj}}+L_{\mathrm{aux}}$$

All model parameters are initialized using Xavier uniform initialization and optimized using the Adam optimizer ($\beta_{1} = 0.9, \beta_{2} = 0.98, \varepsilon= {10}^{-9}$) with the Noam learning rate schedule, which incorporates a linear warmup phase followed by an inverse square-root decay.

**Streaming Inference Strategy**

To satisfy the latency requirements for intraoperative assistance, a streaming inference strategy was implemented in which instrument detection and scene feature extraction were performed online as each video frame arrived and cached in memory. This design masked the processing time of the first 7 frames within the inter-frame interval, such that the effective output latency was determined primarily by processing of the final frame and Transformer inference. On a platform equipped with an AMD Ryzen Threadripper 7960X CPU and an NVIDIA RTX 5090 GPU, preprocessing required 15.3 ms per key frame and end-to-end latency for the final frame was 32.7 ms.

**Evaluation Metrics**

All metrics are computed directly in pixel space and the instrument positions are represented as absolute pixel coordinates on the 1920×1080 image plane throughout the entire pipeline. We report three evaluation metrics to compare the performance of different models (lower is better for all):

Average displacement error (ADE), defined as the mean Euclidean distance between predicted and ground-truth positions across all predicted time steps:

$$\mathrm{ADE} =\frac{1}{T_{\mathrm{pred}}}\sum_{k=1}^{T_{\mathrm{pred}}} \left\| {\hat{\boldsymbol{P}}}_{T_{\mathrm{pred}}}-\boldsymbol{P}_{T_{\mathrm{pred}}} \right\|$$

Final displacement error (FDE), defined as the Euclidean distance at the last predicted time step:

$$\mathrm{FDE} =\left\| {\hat{\boldsymbol{P}}}_{T_{\mathrm{pred}}}-\boldsymbol{P}_{T_{\mathrm{pred}}} \right\|_{2}$$

Discrete Fréchet Distance (FD), evaluating global shape similarity between the predicted and ground-truth trajectories:

$$\mathrm{dp}\left[ i,j \right] =\max\left( \mathrm{dp}\left[ i-1,j \right], \mathrm{dp}\left[ i-1,j-1 \right], \mathrm{dp}\left[ i,j-1 \right], \left\| {\hat{\boldsymbol{P}}}_{i}-\boldsymbol{P}_{j} \right\|_{2} \right)$$

$$FD =dp\left[ T_{\mathrm{pred}}, T_{\mathrm{pred}} \right]$$

**Supplementary Methods 3. Transfer Fine-Tuning for Ex Vivo Environment Adaptation**

Before initiation of the training study, the Scene-Aware Transformer was transfer fine-tuned using 20 previously collected ex vivo porcine kidney suturing videos to adapt the model to the visual and motion characteristics of the training environment. These 20 videos were recorded from 5 experienced urologists performing standardized renal wound suturing on fresh porcine kidneys using the same wound model specifications as described in Methods. The same data pipeline, including suturing scene annotation, phase labeling, and sliding-window trajectory sampling was applied. These recordings were therefore used as expert-style ex vivo trajectories for domain adaptation before the prospective training study.

Transfer fine-tuning was performed by initializing the model with pretrained weights from the in vivo RAPN dataset and continuing training on the ex vivo dataset for 120 epochs with a reduced initial learning rate of 1 x 10^-4^ and the same Noam warm-up schedule. The frozen YOLOv13x backbone was also fine-tuned on 2,400 annotated ex vivo frames to accommodate differences in visual appearance between in vivo and ex vivo settings, including differences in tissue color, texture, lighting, deformation pattern, and background anatomy. After transfer fine-tuning, the model achieved an ADE of 28.6 pixels and FDE of 43.2 pixels on a held-out ex vivo validation set (4 videos), indicating adequate adaptation to the training environment.
